# Redox-stratified macromolecule degradation supports microbial survival in the oligotrophic Kermadec Trench sediments

**DOI:** 10.1038/s41467-026-77249-x

**Published:** 2026-08-25

**Authors:** Yongxin Lv, Peiyu Liu, Yan Liu, Liang Dong, Jinhua Li, Yu Zhang

**Affiliations:** 1 State Key Laboratory of Submarine Geoscience, School of Oceanography, Shanghai Jiao Tong University, Shanghai, China; 2 Key Laboratory of Polar Ecosystem and Climate Change, Ministry of Education, Shanghai Jiao Tong University, Shanghai, China; 3 Shanghai Key Laboratory of Polar Life and Environment Sciences, Shanghai Jiao Tong University, Shanghai, China; 4 Shanghai Changxing Ocean Laboratory, Shanghai, China; 5 Hainan Research Institute, Shanghai Jiao Tong University, Sanya, China; 6 Key Laboratory of Deep Petroleum Intelligent Exploration and Development, Institute of Geology and Geophysics, Chinese Academy of Sciences, Beijing, China; 7 College of Earth and Planetary Sciences, University of Chinese Academy of Sciences, Beijing, China

**Keywords:** Environmental microbiology, Marine biology

## Abstract

Kermadec Trench is a hadal ecosystem in the South Pacific with the water depth 10,047 m. The trench bottom harbors a highly active and populated microbial community, despite the surface sediment is characterized as extremely pressurized, oligotrophic and with low oxygen concentration. It is intriguing, as well as technically challenging, to investigate the microbial adaptation strategies therein. Here we performed the in situ RNA fixation on sediment samples with the assistant of Fendouzhe manned submersible, to approach natural status of microbial metabolisms on both genomic and transcriptomic levels. We reconstructed 1369 metagenome-assembled genomes (MAGs), revealing dominant heterotrophic lineages encoding carbohydrate-active enzymes targeting complex macromolecules such as peptidoglycan and β−1,4-mannan. These degradation processes were transcriptionally coupled with flexible respiratory pathways utilizing oxygen, nitrate, and nitrite as electron acceptors. Co-expression analyses and microbial co-occurrence networks demonstrated niche partitioning driven by redox stratification, with slope communities favoring oxidative pathways and bottom communities enriched in reductive metabolisms, including denitrification and N₂O reduction. Despite compositional divergence, both habitats exhibited conserved functional strategies centered on macromolecule recycling and redox-coupled respiration. Our findings highlight a coordinated system of organic matter remineralization and electron acceptor versatility that underpins microbial survival in Earth’s deepest seafloor ecosystems.

## Abstract (web summary)

Understanding how microbes survive in the crushing pressure and nutrient-poor conditions of the deepest ocean has remained a major challenge. This study reveals that Kermadec Trench microbes thrive by recycling organic matter and flexibly switching energy-generation pathways.

## Introduction

The Kermadec Trench exhibits classic deep-sea trench features, including darkness, extremely high hydrostatic pressure, and a V-shaped morphology^1^. Despite these inhospitable environmental conditions, in situ benthic microbial population size and activity in the Kermadec Trench are elevated compared to adjacent abyssal plains, creating a notable paradox^2–4^. For example, the microbial biomass in the Kermadec Trench’s deepest regions is notably higher due to the increased lateral organic matter transport and accumulation along the axis of the trench^5^. However, the limited knowledge of microbial genetic potential and transcriptional activity restricts our understanding of the adaptive strategy for surviving and maintaining high biomass in such extreme environments.

Located in the South Pacific Ocean, the Kermadec Trench overlies a region of exceptionally low primary productivity (~380 mg C m⁻² d⁻¹), which is substantially lower than that of other well-studied trench systems such as the Atacama (910 mg C m⁻² d⁻¹) and Japan Trenches (760 mg C m⁻² d⁻¹)^2,6,7^. Consequently, the downward flux of particulate organic matter (POM) is limited, and the organic material reaching the trench floor is predominantly composed of recalcitrant macromolecules, such as polysaccharides and peptidoglycan, derived from microbial necromass and degraded planktonic detritus. The total organic carbon (TOC) content of Kermadec sediments (0.21–0.35% wt) is comparable to that of the oligotrophic North Pacific Gyre, highlighting the severe organic carbon limitation in this hadal environment^8,9^. During downward transport through the water column, the more labile fractions are selectively remineralized by heterotrophs, leaving behind an increasingly refractory, structurally complex organic pool that is ultimately delivered to sediments^10–12^.

This oligotrophic stress is compounded by the low oxidative potential of the sediments. Oxygen penetration depth (OPD) in the Kermadec Trench is shallow (~10 cm), despite the limited organic input, and oxygen uptake rates are similar to those measured in more productive trench systems^13–15^. This apparent mismatch suggests that microbial communities may supplement oxygen respiration with alternative electron acceptors such as nitrate, nitrite, or sulfur species, a strategy previously observed in previous studies^16,17^. However, the underlying genomic and transcriptomic ability that enables such metabolic flexibility remains poorly understood. In particular, how microorganisms couple the degradation of refractory macromolecules with the use of diverse terminal electron acceptors in situ remains an open question.

To address these questions, we conducted an integrated genome-resolved investigation using 42 metagenomic and 16 metatranscriptomic datasets from sediments of the Kermadec Trench, including 16 in situ RNA-fixed samples. We uncovered microbial communities with remarkable metabolic versatility, capable of degrading diverse macromolecules and utilizing a broad spectrum of terminal electron acceptors beyond oxygen. Network analyses revealed niche partitioning driven by redox stratification, and co-transcription of macromolecule-degrading and electron-acceptor pathways suggested tight functional coupling. These results shed light on survival strategies in extreme oligotrophic habitats and provide a mechanistic understanding of microbial biogeochemical roles in hadal ecosystems.

## Results

### Diversity and transcriptional activity of microbes in the Kermadec Trench

**Fig. 1:**
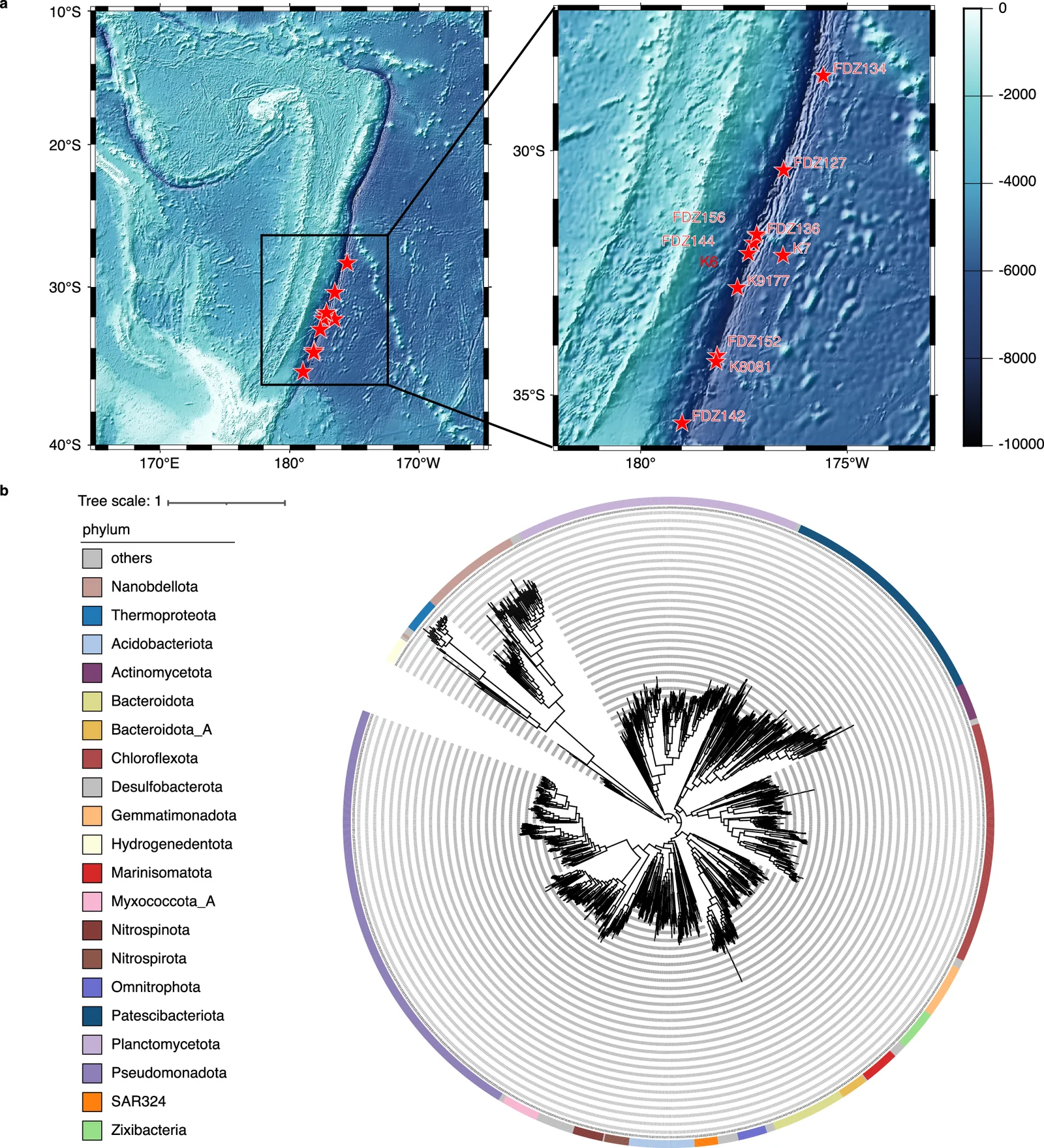
Sampling locations and phylogenetic diversity of representative MAGs from the trench slope sediments. **a** Bathymetric map showing sampling sites along the slope of the trench (left), with a magnified view (right) highlighting individual sample stations. Red stars denote sampling sites, and corresponding labels indicate site codes. **b** Maximum-likelihood phylogenetic tree based on concatenated conserved single-copy marker genes derived from representative MAGs. Outer color ring indicates phylum-level taxonomic affiliation. MAGs classified as “others” include lineages belonging to phyla with less than 10 MAGs.

A total of 16 samples collected from two dives at approximately 10 km water depth were used to generate nine metagenomic datasets and nine metatranscriptomic datasets (Fig. 1a and Supplementary Data 1). Stabilization with RNAlater immediately upon collection ensures that these data provide a faithful representation of in situ microbial community structure and transcriptional activity^18^. Assembly of the metagenomic data produced 25,949,825 contigs (≥1000 bp), which were subsequently binned. After dereplication, we recovered 1369 medium-quality representative MAGs with >50% completeness and <10% contamination, including 464 high-quality MAGs (>90% completeness and <5% contamination)^19^. Classification of the MAGs revealed 99 archaeal and 1250 bacterial MAGs, spanning 5 archaeal and 45 bacterial phyla (Fig. 1b and Supplementary Data 2).

To further assess taxonomic composition, qualified reads were mapped to representative MAGs. The results showed that the most abundant phylum was *Pseudomonadota* (34.41 ± 7.65%), followed by *Thermoproteota* (13.44 ± 10.50%), *Planctomycetota* (9.06 ± 3.11%), and *Chloroflexota* (7.89 ± 3.90%) (Supplementary Fig. 1). We performed unsupervised clustering of the samples based on the RPKM values of MAGs, which revealed a clear spatial separation into two groups: slope (water depth <9 km) and bottom (water depth > 9 km). Based on this grouping, we conducted a non-metric multidimensional scaling (NMDS) analysis, which demonstrated a significant difference between the two groups (Supplementary Fig. 2a; Adonis test, *p* < 0.001). Because our dataset included both newly generated samples and publicly available metagenomes, we further assessed whether the observed slope–bottom separation could be confounded by sample source. NMDS analysis grouped by sample origin showed no significant source-associated separation (Supplementary Fig. 2b; Adonis test, *p* = 0.06).

We assessed the transcriptional activity by identifying actively transcribed genes, with a transcript per million (TPM) value greater than 0.01 in at least five samples. The results revealed that 57.92% (*n* = 1,934,917) of the genes were actively transcribed. We determine the functional role of each MAG based on these actively transcribed genes and their functional annotations (Supplementary Note 1).

### Degradation and utilization of macromolecules

**Fig. 2:**
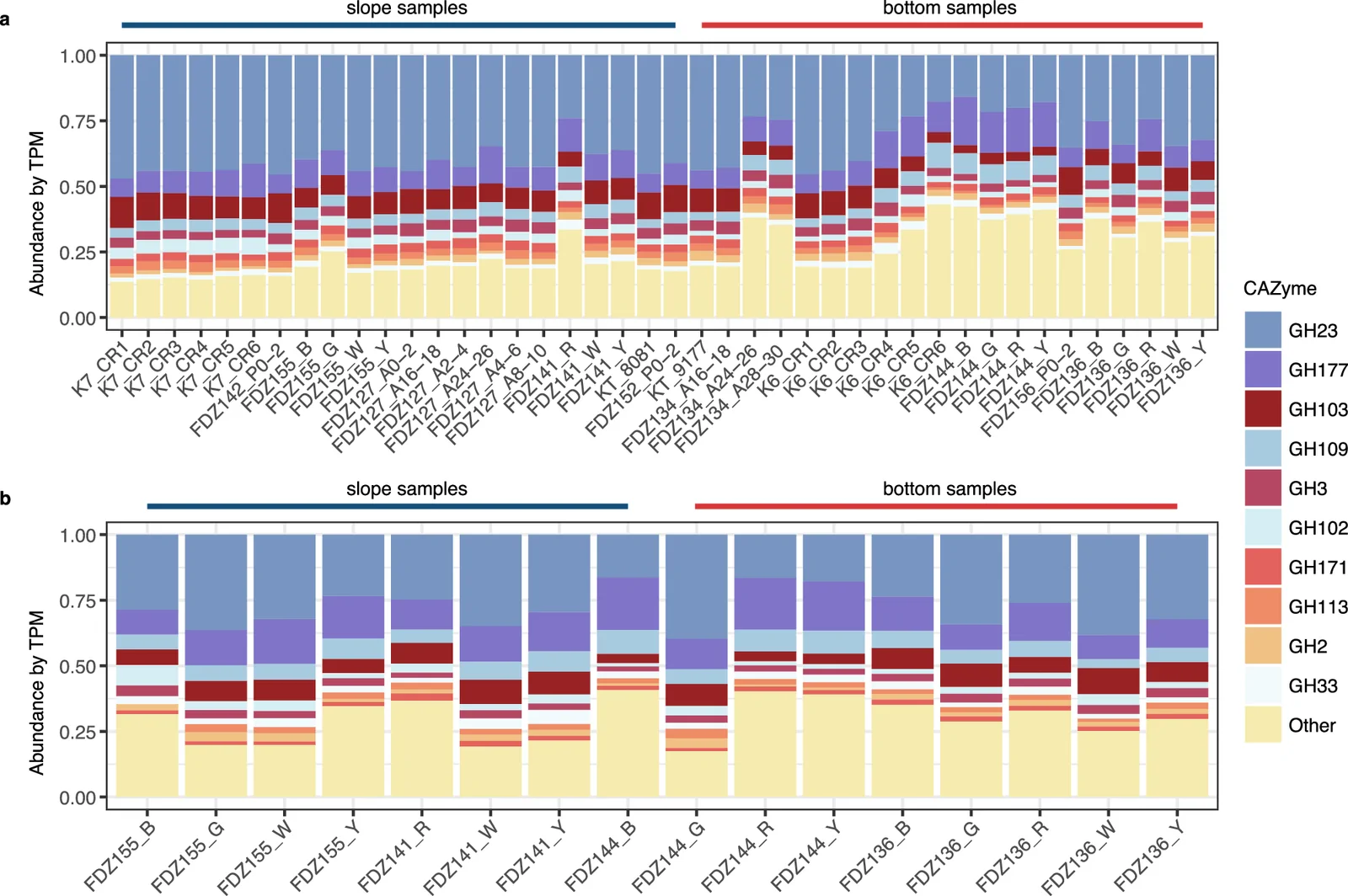
Composition of the abundant CAZyme families across slope and bottom sediment samples. **a** Relative abundance of CAZyme families based on metagenomic data, presented as TPM across all sediment samples. **b** Relative abundance of CAZyme families derived from metatranscriptomic data for a subset of slope and bottom samples. Only the top ten CAZyme gene families in terms of relative abundance are shown, with the remaining grouped as “Other.” Slope and bottom samples are indicated by color-coded horizontal bars above the stacked bar plots. Source data are provided as a Source data file.

We focused on genes encoding extracellular carbohydrate-active enzymes (CAZymes) involved in carbohydrate degradation. A total of 4367 genes were identified, including 4167 glycoside hydrolase (GH) genes and 200 polysaccharide lyase genes. All of the identified genes were actively transcribed. The ten mostly highly expressed families were consistent across both slope and bottom samples, based on the metagenomic and metatranscriptomic datasets. These ten CAZymes contributed accounted for approximately 70% of total CAZymes (75.37 ± 9.05% for metagenomic datasets and 70.49 ± 8.08% for metatranscriptomic datasets) (Fig. 2a, b). We then conducted further analysis based on these 10 CAZymes. The NMDS results of the metatranscriptome indicated that there were no significant differences in the relative abundance of these 10 CAZymes between bottom and slope sediments (Supplementary Fig. 3; Adonis test, *p* = 0.341).

Based on the CAZy database and previous studies, these enzymes can be grouped into two functional categories: two functional groups: those primarily targeting macromolecules such as polysaccharides and peptidoglycan (e.g., GH23, GH103, and GH102) and those targeting low-molecular-weight (LMW) organic substrates (e.g., GH2, GH3, and GH109) (Supplementary Data 3). Enzymes involved in macromolecular degradation generate oligosaccharides and peptides, which can be further broken down into amino acids, smaller oligosaccharides, or monosaccharides. These breakdown products are subsequently transported into microbial cells to support growth or energy production (Supplementary Note 2 and Supplementary Fig. 4). For instance, GH113 degrades β−1,4-mannan into manno-oligosaccharides^20^, which are then processed by enzymes such as GH2 into glucose and mannose. These monosaccharides can subsequently enter central metabolic pathways such as the pentose phosphate pathway or glycolysis (Supplementary Fig. 5).

Additionally, the TPM of genes related to enzymes involved in macromolecular degradation was significantly correlated with the TPM of genes associated with central carbohydrate metabolism (two-sided Spearman test, *R* = 0.78, *P* < 0.01, Supplementary Fig. 6). Additionally, we analyzed the carbon fixation potential within representative MAGs. Based on key genes and module completeness (Supplementary Note 1), we identified 179 MAGs capable of carbon fixation, and 651 MAGs could use macromolecules. Overall, MAGs with carbon fixation capability accounted for 8.58 ± 0.78% based on metagenomic RPKM values, with 63.08 ± 7.50% representing MAGs capable of utilizing high molecular compounds. These results collectively suggest that heterotrophic microbial communities in the Kermadec Trench sediments are primarily sustained by the degradation of macromolecular organic matter rather than by carbon fixation.

### Correlation between the degradation of macromolecules and utilization of potential electron acceptors

**Fig. 3:**
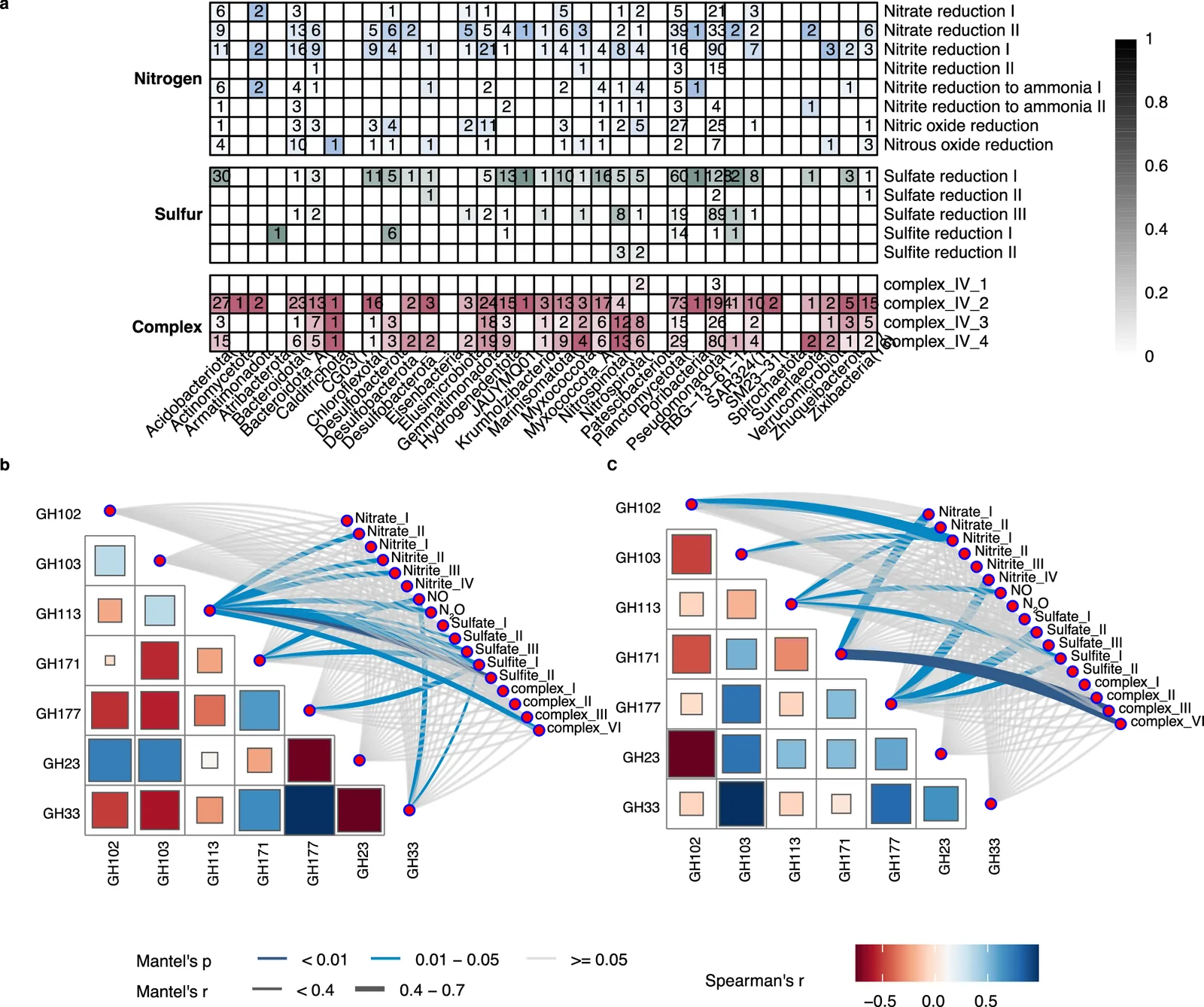
Expression profiles of electron-accepting processes among macromolecule-degrading MAGs and their correlation with CAZyme transcriptional activity. **a** Distribution of active transcripts associated with nitrogen-, sulfur-, and cytochrome complex-related electron-accepting processes across 651 MAGs capable of macromolecule degradation. Numbers within each cell indicate the number of MAGs with active transcription of the corresponding pathway, while color intensity represents the proportion of MAGs within each phylum exhibiting transcriptional activity for that process. **b**, **c** Mantel test analysis evaluating the correlation between the expression of CAZymes and electron-accepting reactions based on metatranscriptomic data from bottom (B) and slope (C) sediment samples. Correlations were assessed using two-sided Spearman’s rank correlation tests. The related genes for each processes were described in the Supplementary Data 5. Source data are provided as a Source data file.

We next investigated the electron acceptors utilized by these 651 MAGs (Fig. 3a and Supplementary Data 4). Based on actively transcribed genes, we identified that 591 MAGs could use oxygen as an electron acceptor, among which 178, 250, and 379 MAGs could also utilize other electron acceptors, such as nitrate, nitrite, and sulfate, respectively. Among the remaining 60 MAGs, which lacked genes associated with complex IV, 17, 12, and 24 MAGs exhibited active transcription of genes involved in nitrate, nitrite, and sulfate reduction, respectively. In terms of transcriptional activity, genes associated with oxygen accounted for 53.55 ± 2.66% of the total transcription activity, followed by nitrite (24.92 ± 3.65%) and nitrate (12.69 ± 4.14%) (Supplementary Fig. 7a). The primary genes involved were *coxABCD* (complex_II), *nirK* (Nitrite reduction I), and *ccoNOPQ* (complex_IV), which contributed 30.68 ± 5.40%, 18.78 ± 4.67%, and 15.29 ± 1.68%, respectively, to all related genes (Supplementary Fig. 7b).

Although the major electron acceptors were similar, we observed significant differences in the transcriptional activity of genes associated with reduction pathways between the bottom and slope sediment samples (Supplementary Fig. 8a; Adonis test, *p* = 0.043). Consequently, we separated the bottom and slope samples for Mantel test analysis to calculate the correlation between the seven most active CAZymes and electron acceptors (Fig. 3b, c). The results revealed 11 significant correlations in the slope samples, primarily involving GH171, GH113, and GH77, with oxygen, nitrate, and nitrite as the dominant electron acceptors. In contrast, the bottom samples showed a broader range of associated electron acceptors, including N_2_O, with correlations primarily centered around GH113. The specific reduction pathways also varied. For example, oxygen reduction was mediated exclusively by *ccoNOPQ* (complex_IV) in bottom sediments but by both *ccoNOPQ* and *cydAB* (complex_III) in slope sediments. Nitrite reduction likewise shifted from *nirS*-mediated denitrification (nitrite_II) and *nrfAH*-mediated dissimilatory nitrite reduction (nitrite_III) in bottom sediments to *nirK*-mediated denitrification (nitrite_I) and *nirBD*-mediated dissimilatory nitrite reduction in slope sediments. The Pielou’s evenness index further indicated significantly higher diversity in the bottom samples (Supplementary Fig. 8b; Wilcoxon rank-sum test, *P* < 0.001). These findings suggest that, within the Kermadec Trench, electron acceptors other than oxygen also contribute to macromolecule degradation. The slope samples predominantly relied on strongly oxidizing electron acceptors, such as oxygen, nitrate, and nitrite, whereas the bottom samples also utilized electron acceptors with weaker oxidation potentials.

### Different electron acceptor based on the co-occurrence network and whole community

**Fig. 4:**
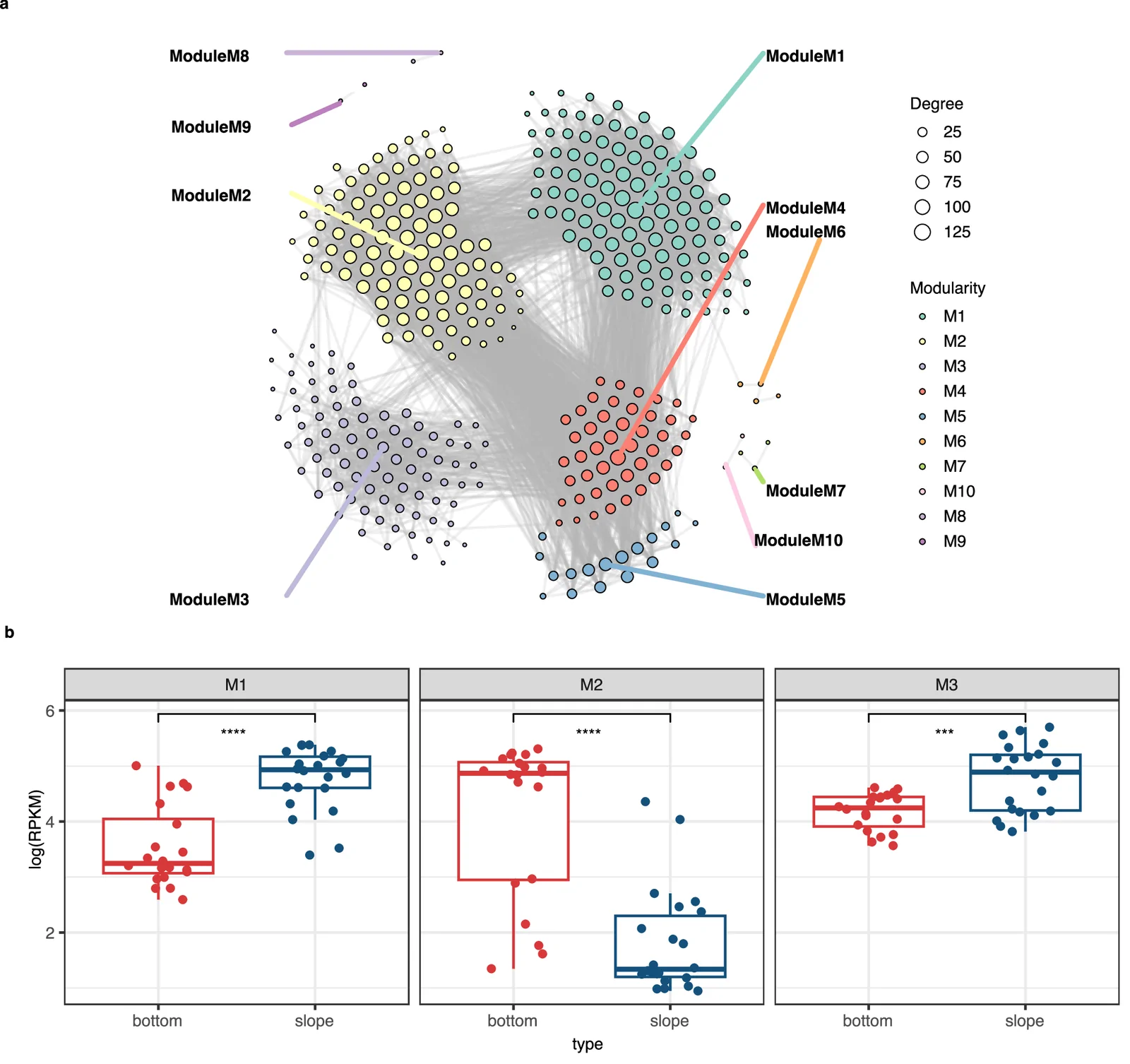
Co-occurrence network structure and habitat-specific abundance of major microbial modules. **a** Microbial co-occurrence network constructed from macromolecule-degrading MAGs. Nodes represent individual MAGs and edges indicate significant co-occurrence relationships. Node size corresponds to degree centrality, and node color indicates module membership determined by network modularity analysis. **b** Relative abundance (log-transformed RPKM) of the three dominant modules (M1, M2, M3) across bottom (*n* = 22) and slope (*n* = 20) sediment samples, based on metagenomic data. No technical replicates were included. Horizontal comparisons were performed using one-sided Wilcoxon rank-sum test. For box plots, the centre line indicates the median, box bounds indicate the 25th and 75th percentiles, whiskers extend to the smallest and largest values within 1.5× the interquartile range, and points represent individual samples. Significance symbols indicate: ***p < 0.001 and ****p < 0.0001. Source data are provided as a Source data file.

Based on RPKM values, we constructed a microbial co-occurrence network (Fig. 4a), which consisted of 359 nodes and 5566 edges. Community detection using the cluster_louvain algorithm identified 10 modules. Considering module size, we focused our subsequent analyses on modules M1, M2, and M3, each containing approximately 100 nodes. According to the relative abundance of MAGs inferred from metagenomic RPKM values, module M2 exhibited significantly higher relative abundance in bottom samples, whereas modules M1 and M3 were significantly enriched in slope samples (Fig. 4b; Wilcoxon rank-sum test, *P* < 0.001). Within these three modules, 80 (73.39%) MAGs in M1, 56 (54.90%) in M2, and 80 (82.47%) in M3 were identified as capable of degrading macromolecular organic matter. Metatranscriptomic analyses based on TPM values revealed that oxygen, nitrite, and nitrate were the predominant terminal electron acceptors across all three modules. Notably, nitrate reduction–related genes exhibited significantly higher TPM values in M2 compared with M1 and M3 (Supplementary Fig. 9a).

Within the same module, when two nodes connected by an edge represented (i) a macromolecule-degrading MAG capable of nitrate consumption and (ii) a MAG capable of nitrate production, we inferred that the latter could serve as an electron acceptor provider for the former. The sources of nitrate and nitrite differed among the three modules, yet similar trends were observed between slope and bottom samples. With respect to nitrate, nitrite oxidation was evident in modules M1 and M3 but was largely absent in M2. For nitrite, ammonia oxidation was more active in M1 and M3 than in M2, whereas denitrification-related TPM values were substantially higher in M2 than in M1 and M3, a pattern that was particularly pronounced in bottom samples (Supplementary Fig. 9b). These findings are consistent with the Mantel test results, which indicated stronger oxidative capacity in slope samples, characterized by active electron acceptor oxidation processes, especially nitrite oxidation, whereas reductive processes predominated in bottom samples.

We further assessed, at the whole-community level, whether electron acceptor–associated redox processes differed between bottom and slope environments. Co-inertia analysis demonstrated that the abundance and TPM of electron acceptor–related genes in metagenomic (RV = 0.8014, *p* < 0.001) and metatranscriptomic (RV = 0.4330, *p* = 0.026) datasets were significantly correlated with microbial community composition (Supplementary Fig. 10).

## Discussion

### Macromolecule depolymerization and electron-acceptor flexibility support hadal microbial life

Through genome-resolved analyses of 42 metagenomes and 16 metatranscriptomes, we investigated the survival strategies employed by microbial communities inhabiting the oligotrophic sediments of the Kermadec Trench. Previous studies have reported diverse pressure-adaptation strategies in piezophilic and piezotolerant microorganisms, such as accumulating compatible solutes (e.g., trimethylamine *N*-oxide or glycine betaine) to stabilize cellular components under high hydrostatic pressure, and optimizing specific enzymes to sustain catalytic performance and metabolism^21–23^. In contrast, this study primarily focuses on how trench microorganisms acquire nutrients. Our results indicate that the microbial community is dominated by heterotrophic microorganisms that are adapted to oligotrophic environments by degrading macromolecules. The microbial hydrolysis therein primarily targets peptidoglycan, sialylated polysaccharides, and β−1,4-mannan (Fig. 2, Supplementary Figs. 5, 6 and Supplementary Note 2), which are directly derived from cellular decay and sinking detritus. These compounds are derived from a range of biological sources associated with both pelagic and benthic processes. Peptidoglycan is a structural polymer that constitutes a major component of bacterial cell walls, and thus accumulates in sediments as a result of microbial cell turnover^24,25^. Sialylated polysaccharides are mainly derived from the remains of sinking phytoplankton and molluscan shell debris^26,27^. Extracellular polymeric substances (EPS), including mannans, are secreted by deep-sea bacteria and archaea as part of biofilm formation and surface colonisation^28,29^.

Microorganisms efficiently scavenge external cellular debris, bypassing the energy-intensive de novo synthesis of complex components^30^. By degrading these materials into simpler intermediates, microbes conserve both energy and nutrients, and can directly reincorporate recycled building blocks into essential structures such as the cell envelope and cytoskeletal elements. This recycling strategy likely enhances survival in resource-limited environments and provides a competitive advantage in ecological niches^31,32^. For example, *Pseudomonas aeruginosa* can utilize peptidoglycan fragments to regenerate its cell wall, while *Planctomycetes* species degrade polysaccharides for energy in oligotrophic environments^33,34^. Similarly, Bacteroidetes in Mariana Trench hadal sediments thrive by deploying hemicellulose-degrading CAZymes to convert complex polysaccharides (including β-mannan) into assimilable sugars for cellular processes^35^.

Beyond oligotrophy, the Kermadec Trench also represents a low-oxidizing-power environment. This is supported not only by the shallow penetration depths of oxygen and nitrate reported previously^14^, but also by our identification of actively transcribed MAGs associated with anaerobic respiration, such as the anaerobic ammonium-oxidizing bacterium (Supplementary Data 4). Consistent with this, the actively expressed gene repertoire in these MAGs includes not only oxygen-related Complex IV components, but also genes involved in nitrate and nitrite reduction (Fig. 3a). Mantel test further revealed significant correlations between CAZymes and reductases across 651 MAGs capable of macromolecule degradation (Fig. 3b). In addition, microbial co-occurrence network analysis highlighted the interaction between MAGs capable of degrading macromolecules and MAGs able to produce nitrite or nitrate. This suggests that microbes utilize alternative electron acceptors besides oxygen in the Kermadec Trench. This inference aligns with culture-based evidence. Roothans and colleagues showed that heterotrophic microorganisms can use nitrate or nitrite as terminal electron acceptors for respiratory degradation of complex organic substrates even under fully oxic conditions (>6.5 mg O₂/L)^36^. Moreover, increasing studies indicate that high hydrostatic pressure may induce intracellular oxidative stress^37,38^, potentially stimulating the microorganisms to utilize alternative electron acceptors^16,38^. For example, studies in the Mariana Trench have detected denitrification processes in surface sediments under oxic conditions^17^, and Yang et al. demonstrated that high pressure can promote microbial utilization of nitrate^39^. Together, these observations suggest that reliance on alternative electron acceptors is likely amplified in the Kermadec Trench, thereby reducing microbial dependence on oxygen and expanding the metabolic options available for organic matter remineralization in hadal environments^39–42^.

### Redox stratification drives niche differentiation in hadal sediments

In addition to the taxonomic differences, we observed pronounced disparities in microbial community structure and electron acceptor profiles between slope and bottom sediments in the Kermadec Trench (Supplementary Figs. 1–2, and 8b). Similar spatial differentiation has been reported in the Mariana Trench, where it has been attributed to the trench’s V-shaped topography causing uneven accumulation of particulate organic matter (POM)^43–45^. This geomorphological feature promotes POM deposition towards the trench axis, leading to accelerated depletion of terminal oxidants such as O₂ and NO₃⁻ in bottom sediments^5,46^. As a result, microbial communities inhabiting the slope exhibit enhanced transcriptional activity related to oxidative metabolisms such as ammonia oxidation, whereas bottom-dwelling communities are more enriched in genes associated with nitrate reduction and anaerobic respiration, reflecting their adaptation to a more reductive environment^13^. Our findings are consistent with these trends. Co-occurrence network analysis revealed that slope-enriched modules (M1, M3) are transcriptionally active for genes involved in ammonia and nitrite oxidation (Fig. 4 and Supplementary Fig. 9b). Furthermore, we detected notable differences in the terminal electron acceptors utilized during macromolecule degradation. Slope communities preferentially rely on stronger oxidants such as nitrate and nitrite, whereas bottom communities appear to additionally exploit NO and N₂O as alternative acceptors (Fig. 3b, c and Supplementary Fig. 8b).

A marked divergence was also found in the expression of genes encoding oxygen-reducing complexes. Mantel test analysis indicated that bottom communities primarily expressed the high-affinity *cbb3*-type cytochrome c oxidase (Fig. 3b), which is known to confer a selective advantage under microoxic conditions^47^. In contrast, slope communities also expressed the *bd*-type oxidase, which is associated with enhanced resistance to oxidative and nitrosative stress^48,49^, and its expression was significantly correlated with the transcriptional profile of macromolecule degradation pathways (Fig. 3c). Together, these observations suggest that microbial communities on the trench slope experience more oxidizing conditions than those in the trench bottom.

Although genome-resolved metagenomic and metatranscriptomic analyses provide valuable insights into the metabolic potential and transcriptional activity of sediment microbial communities, this omics-based approach has inherent limitations. Metabolic pathways were inferred from reconstructed MAGs, some of which are incomplete; thus, the absence of specific genes should not be taken as definitive evidence for the absence of corresponding functions. Importantly, the lack of paired geochemical measurements from the same sediment horizons limits direct assessment of porewater chemistry, redox gradients, electron acceptor availability, and process rates. These limitations preclude us from directly linking specific metabolic potentials or transcript patterns to measured in situ redox conditions, electron acceptor availability, or quantitative biogeochemical fluxes.

Collectively, we propose a conceptual model of microbial adaptation in the sediments of the Kermadec Trench, focusing on stresses mainly caused by the oligotrophic condition rather than hydrostatic pressure. Variability in POM accumulation between slope and bottom regions shapes the local redox environment, driving distinct preferences for terminal electron acceptors and, in turn, divergent community compositions (Supplementary Fig. 10). Despite this metabolic divergence, the communities exhibit functional convergence through broadly similar repertoires of CAZymes, supporting survival under extreme nutrient limitation. Moreover, they facilitate sustained organic matter remineralization by coupling heterotrophic degradation to the use of alternative electron acceptors such as nitrite and nitrate, representing a clear deviation from canonical aerobic respiration. These findings deepen our understanding of hadal microbial ecology and suggest that similar couplings between macromolecule degradation and flexible respiratory strategies may underpin biogeochemical cycling across other deep-sea ecosystems. The future studies are to perform direct monitoring on the in situ geochemistry and microbial activity to provide the quantitative evaluation on the ecological impact of these processes.

## Methods

### Sampling sites, DNA/RNA extraction and sequencing

Sixteen sediment samples were collected from the Kermadec Trench during cruise TS29 in 2022. Approximal 300 ml surface sediments (0–10 cm below seafloor) were collected and fixed in situ with 360 mL RNAlater using a deep-sea sediment RNA fixation sampler operated by the Fendouzhe manned submersible. The sediment sample was collected by push core and injected into the upper chamber of this deep-sea sediment RNA fixation sampler, in which the RNAlater was preloaded in the lower chamber. Afterwards, the sampler was closed, and the partition between two chambers was removed by the submersible manipulator. The RNAlater was prepared according to the recipe (dx.doi.org/10.17504/protocols.io.c56y9d). After the samples arrived on board, they were stored at −20 °C until sequencing. The methods of extraction and sequencing for DNA and RNA were described in the Supplementary Note 3. Besides these 16 samples, we also collected 26 published metagenomic datasets from Kermadec Trench for analysis^50–53^. The sampling sites for all datasets were visualized by GMT^54^.

### Bioinformatic workflow for omics data analysis

Metagenomic and metatranscriptomic data were processed to assess microbial community function and transcriptional activity, following methodologies consistent with previous studies^39,55^. After quality control and assembly, we reconstructed metagenome-assembled genomes (MAGs) and retained high-quality, non-redundant MAGs (completeness >50%, contamination <5%) for downstream analysis. Genes were predicted and functionally annotated for KEGG Orthology (KO) and CAZy families. Secretion signals for CAZymes and peptidases were predicted to infer extracellular enzymes. For metatranscriptomes, rRNA-depleted reads were mapped to the gene catalog for quantification of transcript abundance. Detailed methods are provided in the Supplementary Note 4–5, Supplementary Fig. 11–13, and Supplementary Data 5–6.

### Co-occurrence network

Microbial co-occurrence networks were constructed using the microeco R package (https://github.com/ChiLiubio/microeco, version 2.0.0). MAGs present in at least 20% of all samples and reaches a relative abundance of at least 0.5% in at least one sample were selected to construct the netwok. Spearman correlation coefficients were calculated among MAGs, and only significant correlations (*P* < 0.05) were retained. To determine an appropriate correlation threshold and minimize noise, we applied the Random Matrix Theory approach, which identified a threshold of 0.81. An undirected weighted network was built based on correlations above this threshold. Modules within the network were detected using the Louvain algorithm (cluster_louvain). Network visualization was performed using the ggnetview package, allowing clear representation of network topology and module structure.

### Statistical analyses and visualizations

All the statistical analyses and visualizations were performed via R (http://www.rproject.org/, version 4.5.2). The Mantel test was analyzed via the linkET package (https://github.com/Hy4m/linkET, version 0.1.0). The Spearman test was analyzed via the ggpubr package (https://rpkgs.datanovia.com/ggpubr/, version 0.6.2). Visualization was performed via the ggplot2 package (https://github.com/tidyverse/ggplot2 version 4.0.1). NMDS was performed using the metaMDS function from the vegan package (https://github.com/vegandevs/vegan, version 2.7-2). Wilcoxon rank-sum test was implemented via the wilcox.test function in base R. Co-inertia analysis was conducted using the coinertia function from the ade4 package (https://github.com/adeverse/ade4, version 1.7-23).

## Supplementary information

Supplementary Information

Description of Additional Supplementary Files

Supplementary Data 1-6

Transparent Peer Review file

## Source data

Source Data

## Data Availability

The metagenomic and metatranscriptomic sequencing data generated in this study have been deposited in the Genome Sequence Archive (GSA) of the National Genomics Data Center (NGDC) under accession number CRA022127, associated with BioProject PRJCA034948. The MAGs generated in this study have been deposited in the Genome Warehouse (GWH) of the NGDC under accession numbers GWHJRBT00000000.1 through GWHJTBP00000000.1 and are publicly accessible through BioProject PRJCA034948. An additional copy of the MAG dataset is available through Figshare [https://doi.org/10.6084/m9.figshare.31203940]. Published datasets used in this study are available from NCBI under accession code PRJNA1111327, PRJEB57745, PRJNA405471 and PRJNA1216632. Source data are provided with this paper.

## Code availability

The custom scripts are available at https://github.com/Gu2uMo/custom-script.

## References

[1] Angel, M. V. Ocean trench conservation. *Environmentalist* **2**, 1–17 (1982).

[2] Glud, R. N. et al. Hadal trenches are dynamic hotspots for early diagenesis in the deep sea. *Commun. Earth Environ.* **2**, 21 (2021).

[3] Xiao, W. et al. Strong linkage between benthic oxygen uptake and bacterial tetraether lipids in deep-sea trench regions. *Nat. Commun.* **15**, 3439 (2024). 10.1038/s41467-024-47660-3 PMC11039702 38653759

[4] Nunnally, C. C., Friedman, J. R. & Drazen, J. C. In situ respiration measurements of megafauna in the Kermadec Trench. *Deep Sea Res. Part I Oceanogr. Res. Pap.* **118**, 30–36 (2016).

[5] Ichino, M. C. et al. The distribution of benthic biomass in hadal trenches: a modelling approach to investigate the effect of vertical and lateral organic matter transport to the seafloor. *Deep Sea Res. Part I Oceanogr. Res. Pap.* **100**, 21–33 (2015).

[6] Schauberger, C. et al. Spatial variability of prokaryotic and viral abundances in the Kermadec and Atacama Trench regions. *Limnol. Oceanogr.* **66**, 2095–2109 (2021). 10.1002/lno.11711 PMC8248377 34239169

[7] Jamieson, A. J., Stewart, H. A., Rowden, A. A. & Clark, M. R. Geomorphology and benthic habitats of the Kermadec Trench, Southwest Pacific Ocean. in *Seafloor Geomorphology as Benthic Habitat* 949–966 (Elsevier, 2020).

[8] Yang, Z. et al. Reliability assessment and paleo-oceanographic signals of geochemical and isotopic indicators in the North Pacific Subtropical Gyre. *Mar. Geol.* **446**, 106777 (2022).

[9] Xu, Y. et al. Distribution, source, and burial of sedimentary organic carbon in kermadec and atacama trenches. *JGR Biogeosci.* **126**, e2020JG006189 (2021).

[10] Xu, C. et al. Non-photosynthetic chemoautotrophic CO_2_ assimilation microorganisms carbon fixation efficiency and control factors in deep-sea hydrothermal vent. *Sci. Total Environ.* **862**, 160805 (2023). 10.1016/j.scitotenv.2022.160805 36502982

[11] Jiao, N. et al. The microbial carbon pump and climate change. *Nat. Rev. Microbiol.* **22**, 408–419 (2024). 10.1038/s41579-024-01018-0 38491185

[12] Veuger, B., Van Oevelen, D., Boschker, H. T. S. & Middelburg, J. J. Fate of peptidoglycan in an intertidal sediment: An in situ^13^ C-labeling study. *Limnol. Oceanogr.* **51**, 1572–1580 (2006).

[13] Wenzhöfer, F. et al. Benthic carbon mineralization in hadal trenches: assessment by in situ O_2_ microprofile measurements. *Deep Sea Res. Part I Oceanogr. Res. Pap.* **116**, 276–286 (2016).

[14] Schauberger, C. et al. Microbial community structure in hadal sediments: high similarity along trench axes and strong changes along redox gradients. *ISME J.* **15**, 3455–3467 (2021). 10.1038/s41396-021-01021-w PMC8629969 34103697

[15] Luo, M. et al. Benthic carbon mineralization in hadal trenches: insights from in situ determination of benthic oxygen consumption. *Geophys. Res. Lett.* **45**, 2752–2760 (2018).

[16] Ling, H., Lv, Y., Zhang, Y., Zhou, N.-Y. & Xu, Y. Widespread and active piezotolerant microorganisms mediate phenolic compound degradation under high hydrostatic pressure in hadal trenches. *Mar. Life Sci. Technol.* **6**, 331–348 (2024). 10.1007/s42995-024-00224-2 PMC11136905 38827128

[17] Nunoura, T. et al. Molecular biological and isotopic biogeochemical prognoses of the nitrification-driven dynamic microbial nitrogen cycle in hadopelagic sediments. *Environ. Microbiol.* **15**, 3087–3107 (2013). 10.1111/1462-2920.12152 23718903

[18] He, Y., Zhang, H., Baltar, F. & Wang, Y. Transcriptional difference of deep-sea microorganisms under different sampling methods. *Environ. Sci. Technol.* **59**, 11653–11665 (2025). 10.1021/acs.est.4c05624 PMC12178249 40465356

[19] The Genome Standards Consortium et al. Minimum information about a single amplified genome (MISAG) and a metagenome-assembled genome (MIMAG) of bacteria and archaea. *Nat. Biotechnol*. **35**, 725–731 (2017). 10.1038/nbt.3893 PMC6436528 28787424

[20] Couturier, M. et al. Functional exploration of the glycoside hydrolase family GH113. *PLoS ONE* **17**, e0267509 (2022). 10.1371/journal.pone.0267509 PMC9032380 35452491

[21] Davydov, D. R., Sineva, E. V., Davydova, N. Y., Bartlett, D. H. & Halpert, J. R. CYP261 enzymes from deep sea bacteria: a clue to conformational heterogeneity in cytochromes P450. *Biotechnol. Appl. Biochem.* **60**, 30–40 (2013). 10.1002/bab.1083 PMC3900297 23586990

[22] Yancey, P. H. Organic osmolytes as compatible, metabolic and counteracting cytoprotectants in high osmolarity and other stresses. *J. Exp. Biol.* **208**, 2819–2830 (2005). 10.1242/jeb.01730 16043587

[23] Scoma, A. & Boon, N. Osmotic stress confers enhanced cell integrity to hydrostatic pressure but impairs growth in Alcanivorax borkumensis SK2. *Front. Microbiol*. **7**, 729 (2016). 10.3389/fmicb.2016.00729 PMC4870253 27242746

[24] Manea, E. et al. Viral infections boost prokaryotic biomass production and organic c cycling in hadal trench sediments. *Front. Microbiol.* **10**, 1952 (2019). 10.3389/fmicb.2019.01952 PMC6716271 31507564

[25] Dang, Y.-R. et al. Phytoplankton-derived polysaccharides and microbial peptidoglycans are key nutrients for deep-sea microbes in the Mariana Trench. *Microbiome* **12**, 77 (2024). 10.1186/s40168-024-01789-x PMC11044484 38664737

[26] Wang, F.-Q. et al. Particle-attached bacteria act as gatekeepers in the decomposition of complex phytoplankton polysaccharides. *Microbiome* **12**, 32 (2024). 10.1186/s40168-024-01757-5 PMC10877868 38374154

[27] Yang, Y. et al. Metagenomic and metatranscriptomic analyses reveal minor-yet-crucial roles of gut microbiome in deep-sea hydrothermal vent snail. *Anim. Microbiome* **4**, 3 (2022). 10.1186/s42523-021-00150-z PMC8722025 34980289

[28] Flemming, H.-C. et al. Microbial extracellular polymeric substances in the environment, technology and medicine. *Nat. Rev. Microbiol.* **23**, 87–105 (2025). 10.1038/s41579-024-01098-y 39333414

[29] Youssef, N. H. et al. In Silico analysis of the metabolic potential and niche specialization of candidate phylum ‘latescibacteria’ (WS3). *PLoS ONE* **10**, e0127499 (2015). 10.1371/journal.pone.0127499 PMC4454575 26039074

[30] Gisin, J., Schneider, A., Nägele, B., Borisova, M. & Mayer, C. A cell wall recycling shortcut that bypasses peptidoglycan de novo biosynthesis. *Nat. Chem. Biol.* **9**, 491–493 (2013). 10.1038/nchembio.1289 23831760

[31] Mayer, C. Bacterial cell wall recycling. in *Encyclopedia of Life Sciences*. 10.1002/9780470015902.a0021974 (Wiley, 2012).

[32] Batuecas, M. T. & Hermoso, J. A. Macromolecular machineries in cell-wall recycling. in *Encyclopedia of Life Sciences* 1–14 10.1002/9780470015902.a0028397 (Wiley, 2019).

[33] Wang, J.-X. et al. The complete genome sequence of the planctomycetotal bacterium Bremerella sp. P1 with abundant genes involved in polysaccharide degradation. *Mar. Genomics* **76**, 101126 (2024). 10.1016/j.margen.2024.101126 39009497

[34] Borisova, M., Gisin, J. & Mayer, C. Blocking peptidoglycan recycling in *Pseudomonas aeruginosa* attenuates intrinsic resistance to fosfomycin. *Microb. Drug Resistance* **20**, 231–237 (2014). 10.1089/mdr.2014.0036 PMC4050453 24819062

[35] Zhu, X.-Y. et al. Deep-sea Bacteroidetes from the Mariana Trench specialize in hemicellulose and pectin degradation typically associated with terrestrial systems. *Microbiome* **11**, 175 (2023). 10.1186/s40168-023-01618-7 PMC10405439 37550707

[36] Roothans, N. et al. Aerobic denitrification as an N_2_O source from microbial communities. *ISME J.* **18**, wrae116 (2024). 10.1093/ismejo/wrae116 PMC11272060 38913498

[37] Welch, T. J., Farewell, A., Neidhardt, F. C. & Bartlett, D. H. Stress response of Escherichia coli to elevated hydrostatic pressure. *J. Bacteriol.* **175**, 7170–7177 (1993). 10.1128/jb.175.22.7170-7177.1993 PMC206858 8226663

[38] Xiao, X., Zhang, Y. & Wang, F. Hydrostatic pressure is the universal key driver of microbial evolution in the deep ocean and beyond. *Environ. Microbiol. Rep.* **13**, 68–72 (2021). 10.1111/1758-2229.12915 33398931

[39] Yang, N., Lv, Y., Ji, M., Wu, S. & Zhang, Y. High hydrostatic pressure stimulates microbial nitrate reduction in hadal trench sediments under oxic conditions. *Nat. Commun.* **15**, 2473 (2024). 10.1038/s41467-024-46897-2 PMC10951307 38503798

[40] Oger, P. M. & Jebbar, M. The many ways of coping with pressure. *Res. Microbiol.* **161**, 799–809 (2010). 10.1016/j.resmic.2010.09.017 21035541

[41] Li, J., Xiao, X., Zhou, M. & Zhang, Y. Strategy for the adaptation to stressful conditions of the novel isolated conditional piezophilic strain Halomonas titanicae ANRCS81. *Appl. Environ. Microbiol* **89**, e01304–e01322 (2023). 10.1128/aem.01304-22 PMC10057041 36912687

[42] Zhang, Y., Li, X., Bartlett, D. H. & Xiao, X. Current developments in marine microbiology: high-pressure biotechnology and the genetic engineering of piezophiles. *Curr. Opin. Biotechnol.* **33**, 157–164 (2015). 10.1016/j.copbio.2015.02.013 25776196

[43] Hu, A. et al. Microbial communities reveal niche partitioning across the slope and bottom zones of the Challenger Deep. *Environ. Microbiol. Rep.* **16**, e13314 (2024). 10.1111/1758-2229.13314 PMC11291871 39086173

[44] Zhou, Y.-L., Mara, P., Cui, G.-J., Edgcomb, V. P. & Wang, Y. Microbiomes in the Challenger Deep slope and bottom-axis sediments. *Nat. Commun.* **13**, 1515 (2022). 10.1038/s41467-022-29144-4 PMC8938466 35314706

[45] Lv, Y., Zhang, L. & Zhang, Y. Clear niche partitioning of nitrite-oxidizing bacteria from the bottom and the slope of Mariana Trench. *Microbiome* **13**, 208 (2025). 10.1186/s40168-025-02192-w PMC12532881 41102734

[46] Turnewitsch, R. et al. Recent sediment dynamics in hadal trenches: evidence for the influence of higher-frequency (tidal, near-inertial) fluid dynamics. *Deep Sea Res. Part I* **90**, 125–138 (2014).

[47] Pitcher, R. S. & Watmough, N. J. The bacterial cytochrome cbb3 oxidases. *Biochim. Biophys. Acta BBA Bioenerg.* **1655**, 388–399 (2004). 10.1016/j.bbabio.2003.09.017 15100055

[48] Borisov, V. B., Nastasi, M. R. & Forte, E. Cytochrome bd as antioxidant redox enzyme. *Mol. Biol.* **57**, 1077–1084 (2023). 10.31857/S0026898423060034 38062962

[49] Wang, W. et al. Cryo-EM structure of mycobacterial cytochrome bd reveals two oxygen access channels. *Nat. Commun.* **12**, 4621 (2021). 10.1038/s41467-021-24924-w PMC8324918 34330928

[50] Li, Y., Liu, H., Xiao, Y. & Jing, H. Metagenome sequencing and 982 microbial genomes from Kermadec and Diamantina Trenches sediments. *Sci. Data* **11**, 1067 (2024). 10.1038/s41597-024-03902-z PMC11445380 39354003

[51] Trouche, B. et al. Distribution and genomic variation of ammonia-oxidizing archaea in abyssal and hadal surface sediments. *ISME Commun.* **3**, 133 (2023). 10.1038/s43705-023-00341-6 PMC10746724 38135695

[52] Peoples, L. M. et al. Microbial community diversity within sediments from two geographically separated hadal trenches. *Front. Microbiol.* **10**, 347 (2019). 10.3389/fmicb.2019.00347 PMC6428765 30930856

[53] Bai, S. et al. Iron is an important influence of volcanic ash input on the evolution of deep-sea ecosystems. *Microbiol. Spectr.* **13**, e00715–e00725 (2025). 10.1128/spectrum.00715-25 PMC12403892 40748195

[54] Wessel, P. et al. The generic mapping tools version 6. *Geochem. Geophys. Geosyst.* **20**, 5556–5564 (2019).

[55] Lv, Y., Zhang, L., Wang, X. & Zhang, Y. Genomic evidence on the distribution and ecological function of Pseudomonas in hadal zone. *BMC Microbiol.* **25**, 100 (2025). 10.1186/s12866-025-03834-7 PMC11869652 40021978

