# Supplementary material for: Redox-stratified macromolecule degradation supports microbial survival in the oligotrophic Kermadec Trench sediments
Source: Nat Commun. 2026 Aug 25;17:10138. doi: 10.1038/s41467-026-77249-x (PMC13601601; doi:10.1038/s41467-026-77249-x)
Supplement: Supplementary file 1 [file 41467_2026_77249_MOESM1_ESM.pdf]

Supporting Information for

Redox-stratified macromolecule degradation supports microbial survival in the oligotrophic Kermadec Trench sediments

Yongxin Lv[1,2,3,4,5], Peiyu Liu[6], Yan Liu[6], Liang Dong[1,2,3], Jinhua Li[6,7], Yu Zhang[1,2,3,4,5*]

[1]State Key Laboratory of Submarine Geoscience, School of Oceanography, Shanghai Jiao TongUniversity, Shanghai 200030, China

[2]Key Laboratory of Polar Ecosystem and Climate Change, Ministry of Education, Shanghai Jiao Tong University, Shanghai 200030, China

[3]Shanghai Key Laboratory of Polar Life and Environment Sciences, Shanghai Jiao Tong University, Shanghai 200030, China.

[4]Shanghai Changxing Ocean Laboratory, Shanghai 201913, China

[5]Hainan Research Institute, Shanghai Jiao Tong University, Sanya 572024, China

[6]Key Laboratory of Deep Petroleum Intelligent Exploration and Development, Institute of Geology and Geophysics, Chinese Academy of Sciences, Beijing 100029, China.

[7]College of Earth and Planetary Sciences, University of Chinese Academy of Sciences, Beijing 100049, China

[*]Correspondence: Yu Zhang, zhang.yusjtu@sjtu.edu.cn

This PDF file contains:

Supplementary Notes 1-5

Supplementary Figures 1-13

Supplementary References 1-28

Supplementary Notes

Supplementary Note 1. Rules to determine the functional role of each MAG

We applied the following criteria to determine the metabolic potential of MAGs.

Degradation of Macromolecules. The MAG contains an actively transcribed gene annotated as GH23, GH177, GH103, GH102, GH171, GH113 and GH33.

Carbon Fixation. The MAG contains actively transcribed key genes (Supplementary Data 4).

Respiration. The MAG contains at least two actively transcribed genes of the same type of respiratory complex (Supplementary Data 4). Notably, MAGs harboring both aerobic and anaerobic respiratory complexes were classified as facultative anaerobes.

Supplementary Note 2. Microbial utilization of degradation products from macromolecules

To assess the microbial utilization of macromolecular degradation products, we analyzed the transcriptional activity of genes involved in their breakdown. A total of 8,306 peptidase genes were identified, with all actively transcribed. 130 Kos were covered, with the most transcriptionally active being K00681 (gamma-glutamyltranspeptidase / glutathione hydrolase; 8.25% ± 1.16%), K01286 (membrane dipeptidase; 5.69% ± 0.95%), and K01322 (serine-type D-Ala-D-Ala carboxypeptidase; 5.69% ± 0.87%) (Supplementary Fig. 4). These peptidases hydrolyze peptidoglycan fragments into amino acids.

Microbial utilization of amino acids involves multiple pathways: incorporation into biomass, conversion to keto acids via transamination or

deamination for energy or biosynthesis, and decarboxylation to amines for pH regulation, defense, or signaling. This study focused on the latter two pathways. Among these, genes related to decarboxylation showed the highest transcriptional activity (48.82% ± 1.02%), followed by those involved in deamination (26.89% ± 1.35%).

Genes encoding GH2, GH3, and GH109 were also highly expressed (Fig. 2B). These enzymes further hydrolyze oligosaccharides into monosaccharides—such as mannose, galactose, and glucose—as well as N-acetylglucosamine (GlcNAc) and N-acetylgalactosamine (GalNAc).

Supplementary Note 3. Extraction and sequencing of DNA and RNA

The sediment sample stored in RNAlater was collected by centrifugation at 8,000 × *g* for 10 min and washed with PBS buffer. Two grams of RNAlater-free sediment sample was mixed with half weight of 0.1 mm and 0.5 mm glass beads mixture and 6 mL of extraction buffer by low-speed vortexing for 5 min. The mixture was homogenized with MP FastPrep-24 homogenizer (MP Biomedicals, CA, USA) at 4.0 m/s for 20 s with a 2 min interval for two cycles. Then, 60 µl lysozyme (100 mg/ml) and 60 µl proteinase-K (20 mg/ml) were added, and the mixture was incubated for 30 min at 37 °C. Then, 420 µl 20% SDS was added and incubated at 65 °C for 2 h. The supernatant was collected by centrifugation at 10,000 × g for 10 min at room temperature. The extraction was repeated once with residual pellets. The supernatants from the two rounds of extraction were combined and mixed with equal volumes of phenol:chloroform:isoamyl alcohol (24:25:1 [v/v], pH 8.0). The aqueous phase was recovered by centrifugation and then precipitated overnight at 4 °C with a 0.6 volume of isopropyl alcohol and 0.1 volume of 3 M sodium acetate (pH 5.2). The nucleic acid pellet was collected by centrifugation at 15 000 × g for 20 min at 4 °C. The supernatant was removed, and the

nucleic acid pellet was washed twice with precooled 70% ethanol and then dissolved in TE buffer. DNA was further purified with the synchronous coefficient of drag alteration (SCODA) method, performed with the Boreal Genomics' Aurora Nucleic Acid Extraction System (Boreal Genomics, Vancouver, BC), and quantified by a Qubit 3.0 fluorometer with a DNA HS Assay Kit (Thermo Fisher Scientific, MA, USA).

The extraction of RNA from sediment samples immersed in RNAlater were carried out using the RNeasy PowerSoil Total RNA Kit (Qiagen, Germany) according to the manufacturer's instructions, then quantified using a Qubit 4.0 Fluorometer (Invitrogen, Carlsbad, CA, USA). To ensure DNA removal, the RNA extracts were treated with TURBO DNase (Invitrogen, Waltham, MA, USA) as directed by the manufacturer. The purified RNA was converted to cDNA, then the metatranscriptomic library was constructed by using Illumina TruSeq Stranded mRNA LT Sample Prep Kit. Purified DNA or RNA samples were sent to the Shanghai Personal Biotechnology (Shanghai, China) for paired-end sequencing by the HiSeq X Ten platform.

Supplementary Note 4. Detail for metagenomic and metatranscriptomic analysis

Firstly, raw reads were trimmed by fastp (version 0.23.4) with the parameter "-q 20 -u 20 -g -c -W 5 -3 -l 90"[1]. Then, the assembly was performed by using either Megahit (version 1.2.9)[2] with "--presets meta-sensitive" for each dive. Contigs shorter than 1000 bp were discarded. The depth matrix of contigs were performed with Bowtie2 (version 2.4.3) with the samples from the same sampling sites (Supplementary Data 1)[3]. Three binning software programs were used to obtain the primary MAGs via the depth matrix, as the best three for single short-reads assembly, comebin (version 1.0.3)[4], semibin2 (version 2.2.1)[5] and metabinner (version 1.4.4)[6].

Binette (version 1.2.1) was used to integrate the results to calculate an optimized, non-redundant set of MAGs for each sample[7]. Quality and taxonomy were determined by using CheckM2 (version 1.2.2) and GTDB-Tk tools (version 2.4.0) with the GTDB database (version220), respectively[8–10]. MAGs with completeness > 50% and contamination < 5% were dereplicated with dRep (version 3.2.2) with the threshold of 95% by ANImf methods[11]. Skani (version 0.3.1) was performed with default parameter to calculate the average nucleotide identity (ANI) value for each pair of MAGs[12]. The distribution was estimated via reads recruitment. Recruitments were performed using BBmap. Qualified hits were used to compute the RPKM values by coverM (https://github.com/wwood/CoverM, version 0.7.0), which reflect a normalized abundance allowing the comparison across different genomes and metagenomes.

For metagenomic assembly and reference MAGs retrieved from GTDB database, gene prediction was performed by using Prodigal (version 2.6.3), and ORFs with a length smaller than 33 amino acids were trimmed[13]. KO of genes were integrated by KofamScan (version 1.3.0), DIAMOND (version 2.0.14) against UniprotKB database (version 20240124), emapper (version 2.1.7) against EggNOB database (version 5.0.2)[14–18]. The alignment should be longer than 50 % of the query sequence and 50% of the subject sequences. Phylogenetic trees were constructed to differentiate the enzymes with the same KO (Supplementary Note 5 and Supplementary Fig. 11-13). The score of KEGG module was evaluated by a custom script[19]. CAZy of each gene was determined by the recommended result of dbCAN (version 5.1.1)[20], which was identified with at least two methods. Only genes classified as GH or PL were used in downstream analysis. Genes encoding CAZymes and peptidases were analyzed using SignalP (version

6.0) to predict signal peptides, allowing us to determine which enzymes are likely secreted extracellularly.

For metatranscriptomic datasets, raw reads were first tirmmed by fastp for poor-quality base and then filtered by Ribodetector (version 0.3.1) for remove rRNA reads[21,22]. The unaligned mRNA reads were mapped to the representative MAGs by BBmap (version 38.96). Then, featureCounts () was used to count the read number of each gene[23]. Finally, a custom python script was used to calculate TPM value[19,24].

To construct the phylogenetic tree, gene or concatenated genes were aligned and trimmed separately by using MAFFT (version 7.490) and trimAL (version 1.4) with the automated1 model[25,26]. IQTREE (version 2.2.0.3) was used to construct the phylogenetic tree with the TEST model[27]. The tree was visualized by iTOL v6[28]. For the tree in Fig. 1B, 40 single copy gene sets were identified by fetchMG (version 1.2) and the subsequent procedure was the same above (https://github.com/motu-tool/FetchMGs). The details of the sequence number, remaining columns and selected model of each tree are given in Supplementary Data 5.

Supplementary Note 5. Differentiate the gene labeled with the same KO

After gene annotation, a phylogenetic tree was constructed to differentiate the enzymes: nitrate reductase/nitrite oxidoreductase (*narGH*/*nxrAB*), ammonia monooxygenase/ methane monooxygenase (*amoABC*/*pmoABC*), and oxidative/reductive dissimilatory sulfite reductase (*odrsAB*/*rdsrAB*). For the tree in the Supplementary Fig. 11-13, genes were extracted according the result of the annotation. The detail about sequence number, remained columns and selected model of each tree was recorded in Supplementary Data 5. All reference genes for the gene trees have been deposited in Figshare (10.6084/m9.figshare.28253687).

Supplementary Figures

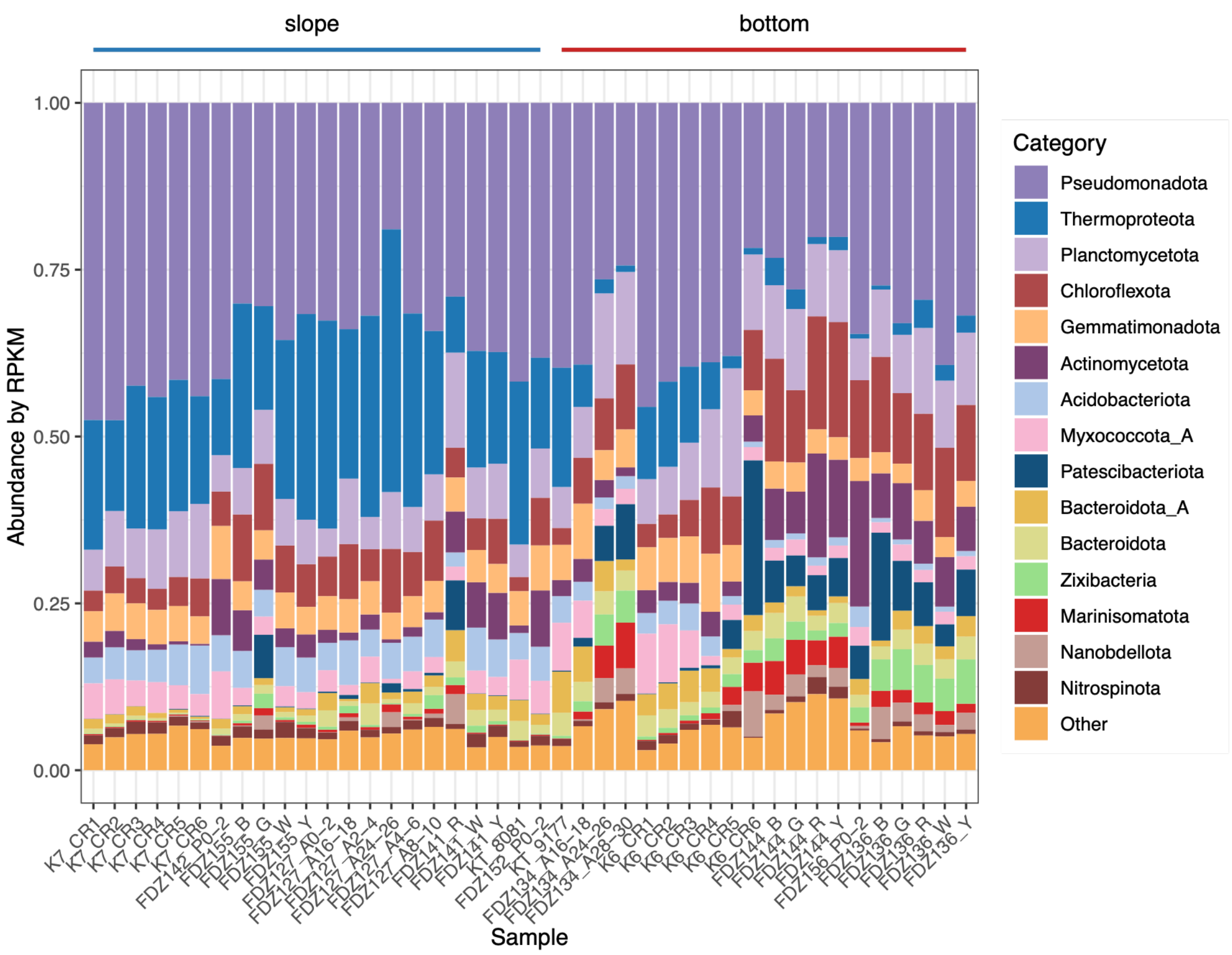

Supplementary Fig. 1

Phylum-level composition of microbial communities across samples. Relative abundances of microbial phyla were calculated based on RPKM-normalized values and are shown as stacked bar plots. Each bar represents an individual sample, with colours indicating different phyla. Samples are grouped by sampling location (slope and bottom). Low-abundance phyla are combined and shown as “Other”.

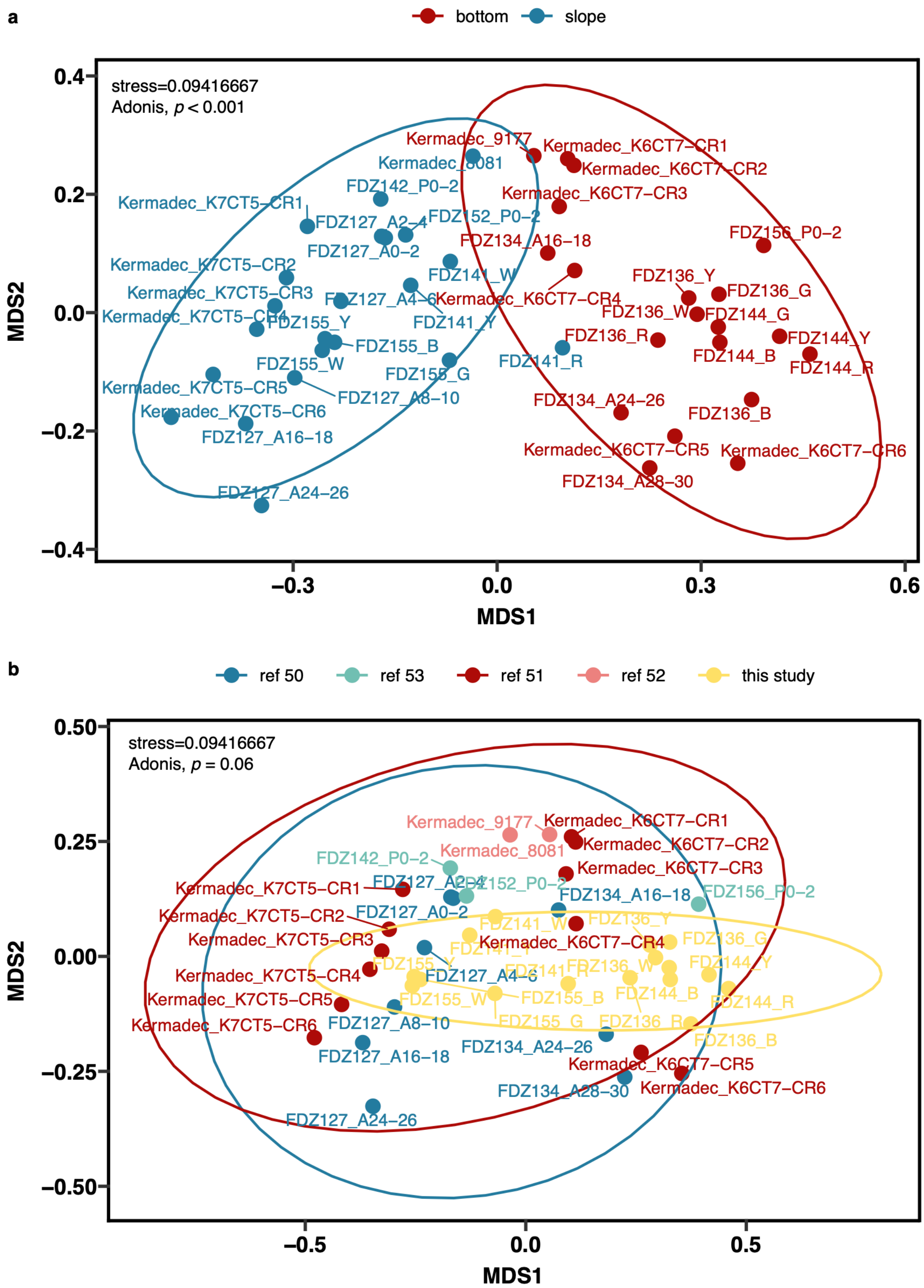

Supplementary Fig. 2

NMDS analysis of microbial community composition. (a) NMDS ordination based on microbial community composition, with samples grouped by seafloor position: slope

(n=20) and bottom (n=22) sites. Each point represents an individual sample, and colors indicate the slope or bottom group. **(b)** NMDS ordination of the same samples grouped by sample source, including publicly available datasets from refs. 50–53 and samples generated in this study. Each point represents an individual sample, and colors indicate different sample sources. Ellipses indicate group-level dispersion.

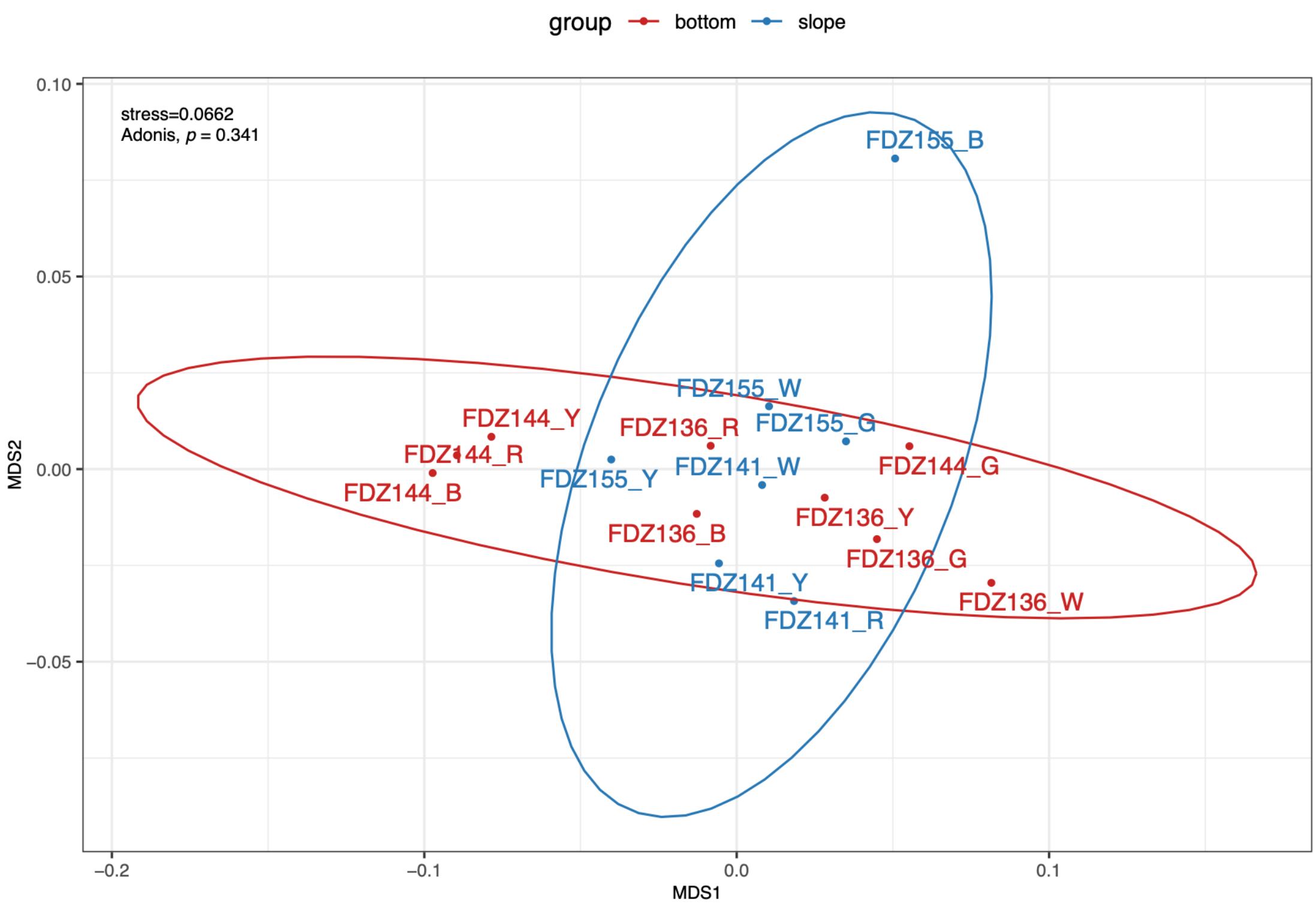

Supplementary Fig. 3

NMDS analysis of CAZyme gene expression profiles in metatranscriptomes. NMDS ordination based on TPM-normalized expression levels of CAZyme genes shows the transcriptional activity patterns of microbial communities from slope (n=20) and bottom (n=22) samples. Each point represents an individual sample, coloured according to sampling location.

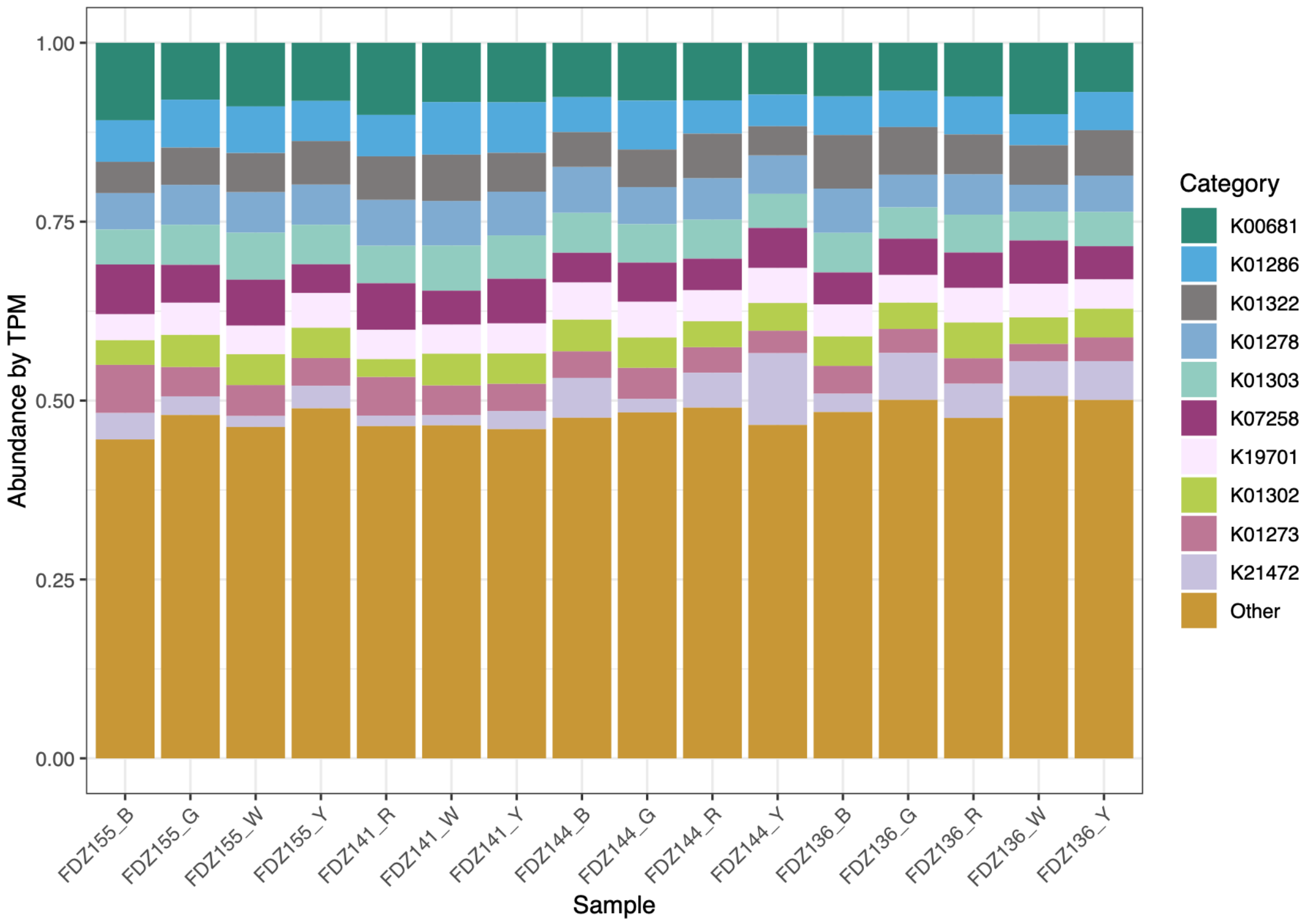

Supplementary Fig. 4

Transcriptional composition of peptidase-related genes across samples. Stacked bar plots show the relative transcriptional abundance of peptidase-related genes based on TPM-normalized metatranscriptomic data. Each bar represents an individual sample, with colours indicating different peptidase KOs. Low-abundance genes are grouped as "Other".

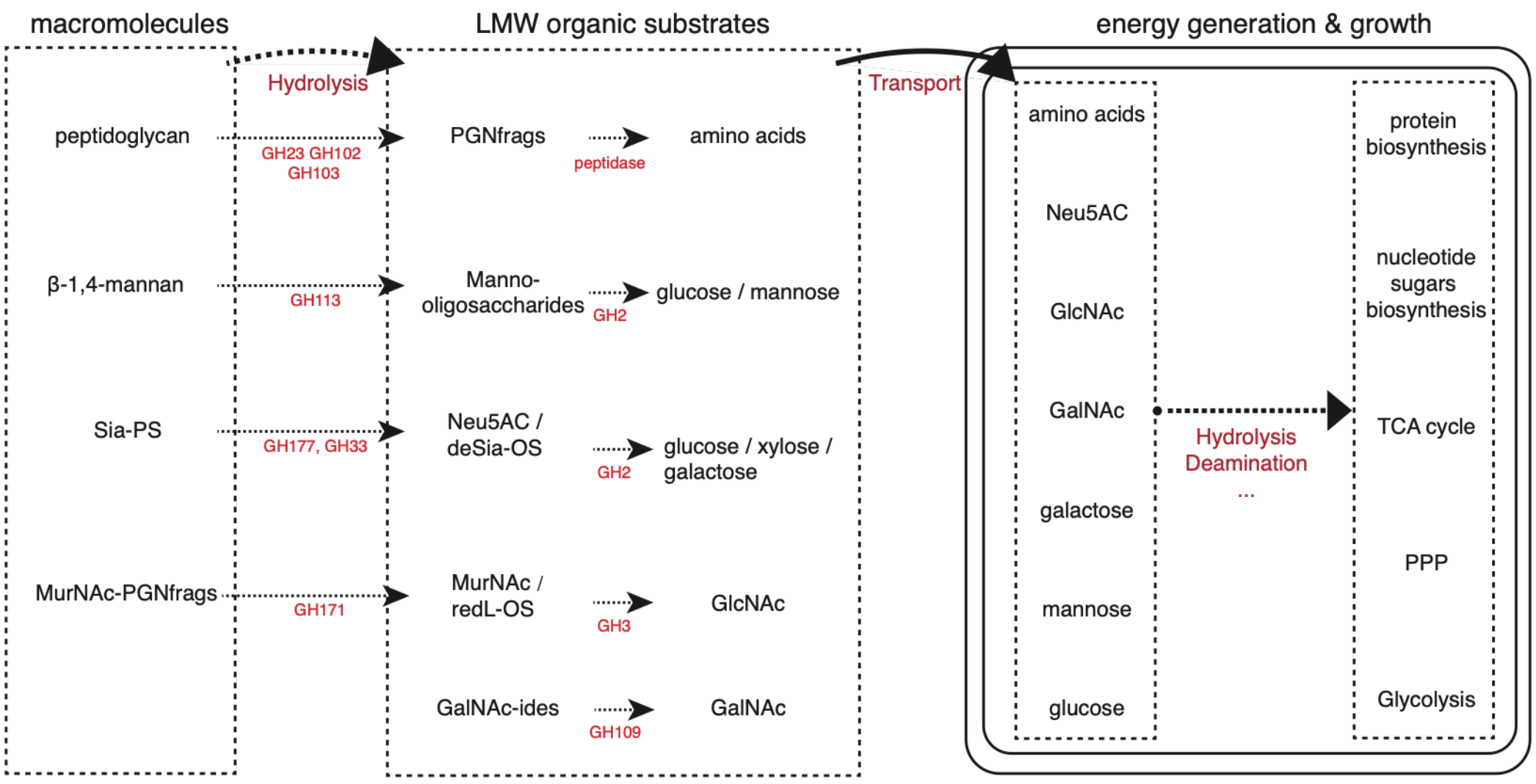

Supplementary Fig. 5

Schematic overview of microbial utilization of macromolecular and LMW organic substrates. LWM = low-molecular-weight; MurNAc-PGNfrags = peptidoglycan fragments with MurNAc; PGNfrags = peptidoglycan fragments; Neu5AC = N-acetylneuraminic acid; deSia-OS = desialylated oligosaccharides; MurNAc = N-Acetylmuramic acid; redL-OS = reduced-length oligosaccharides; GalNAc-ides = N-acetylgalactosaminides; GlcNAc = N-acetylglucosamine; GalNAc = N-acetylgalactosamine.

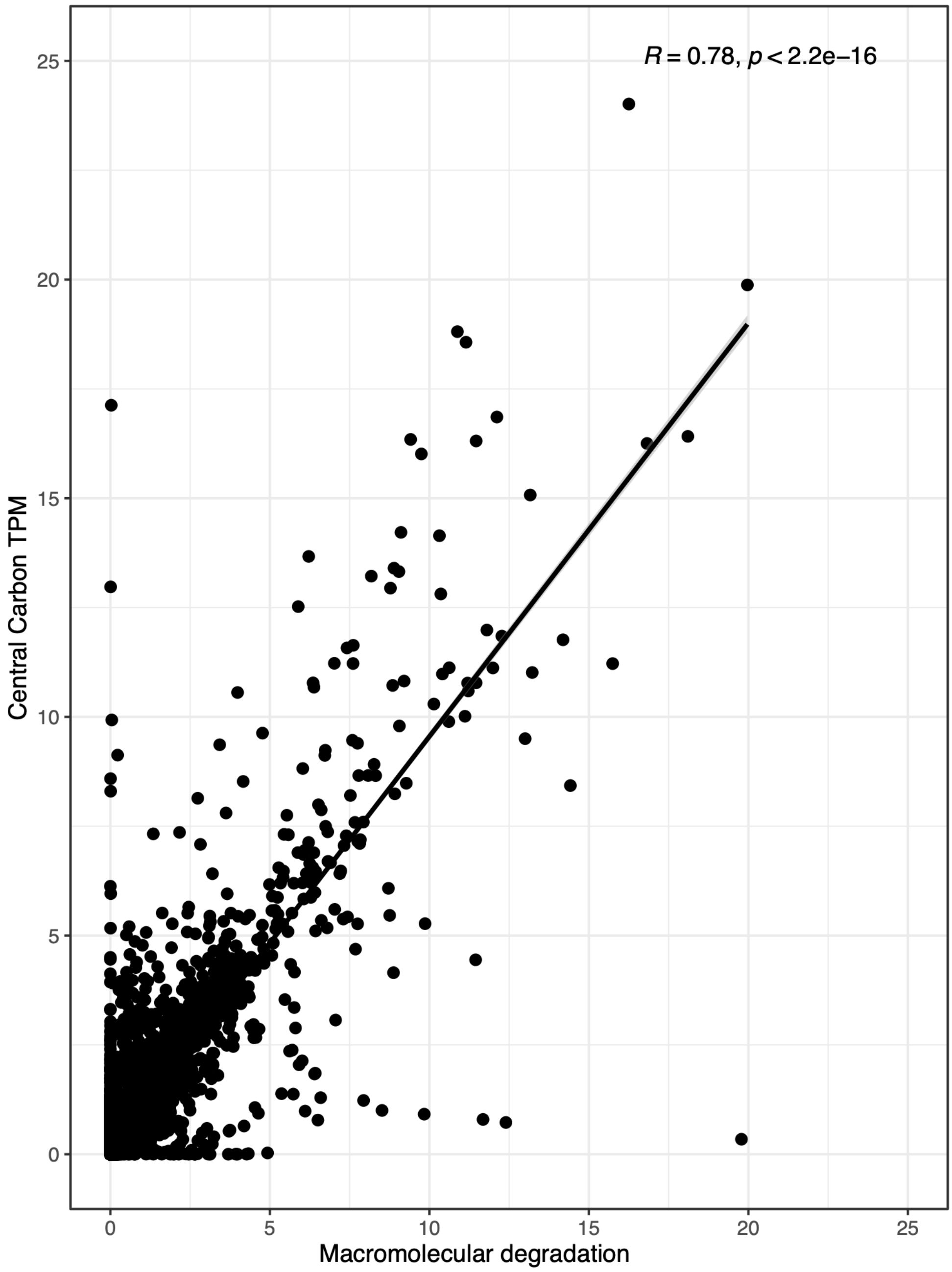

Supplementary Fig. 6

Relationship between macromolecular degradation and central carbon metabolism. Scatter plot showing the correlation between TPM-normalized transcriptional activity of macromolecular degradation pathways and central carbon metabolism. Correlations were assessed using two-sided Spearman's rank correlation test.

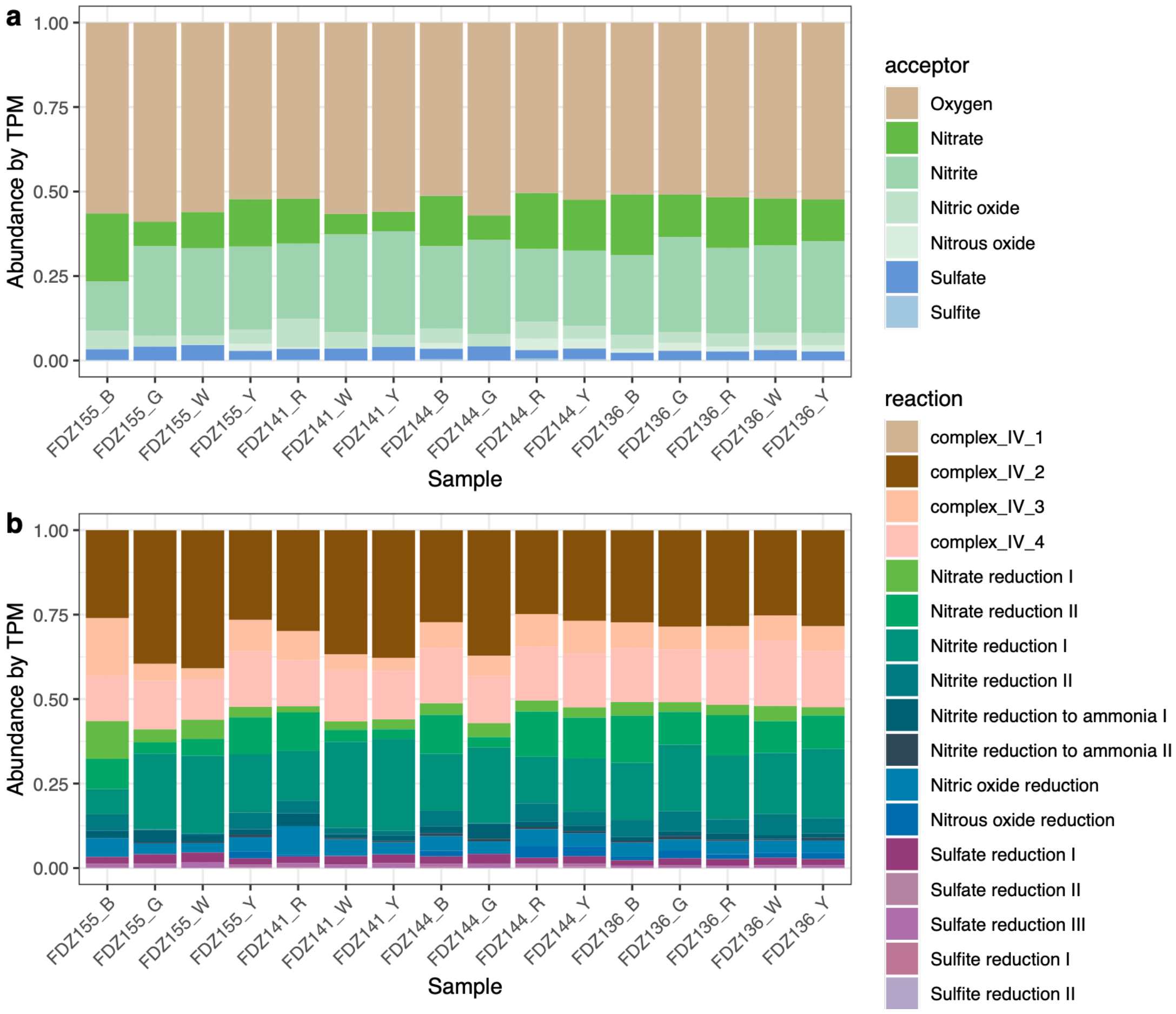

Supplementary Fig. 7

Transcriptional composition of electron acceptors and associated pathways. (a) Relative transcriptional abundance of genes associated with different electron acceptors based on TPM-normalized metatranscriptomic data. **(b)** Relative transcriptional abundance of pathways involved in electron acceptor utilization, including aerobic respiration and nitrogen and sulfur reduction processes. Stacked bar plots represent individual samples, with colours indicating different electron acceptors (a) or metabolic pathways (b).

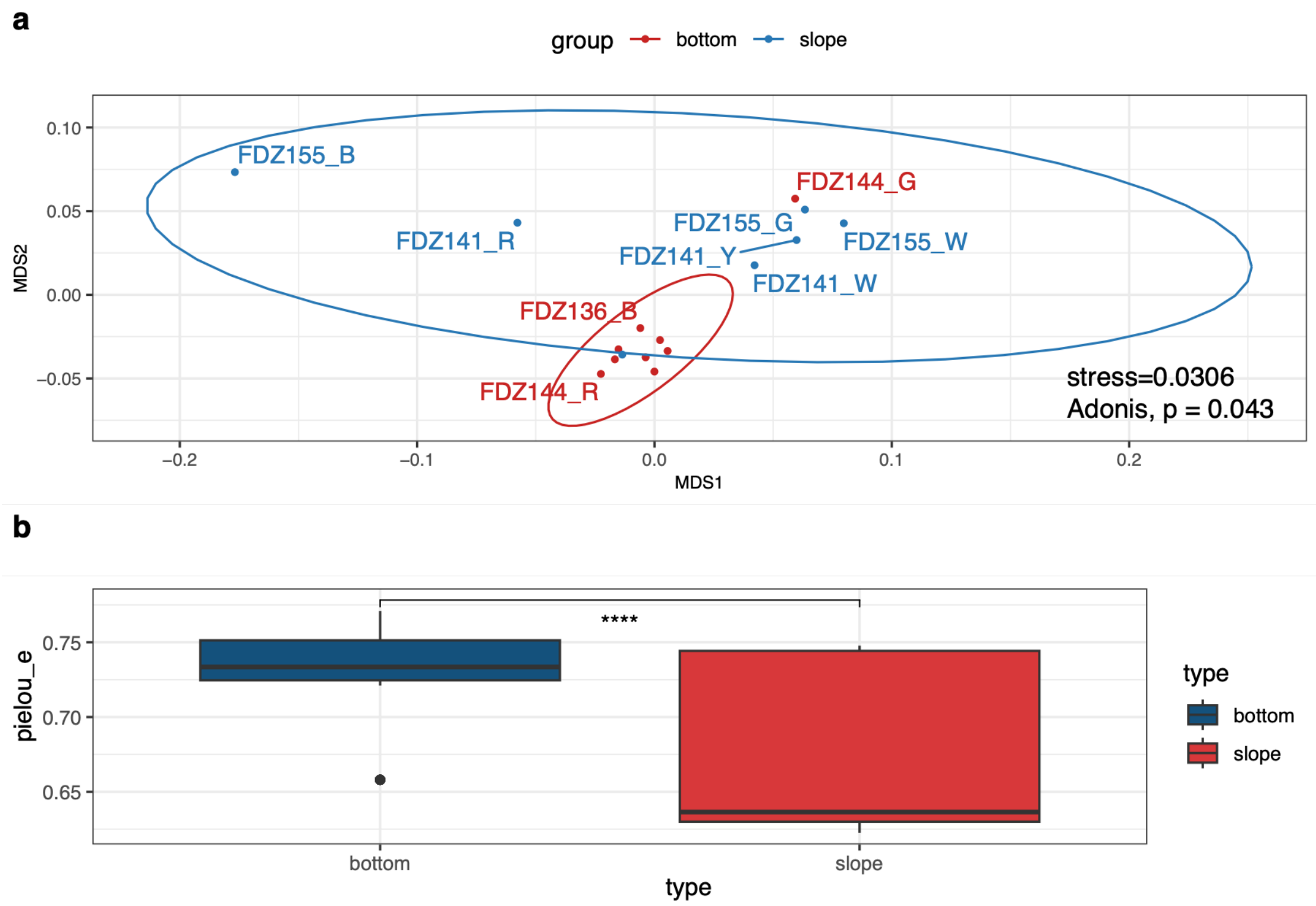

Supplementary Fig. 8

Transcriptional profiles of electron acceptor–related genes in macromolecule-degrading MAGs. Analyses were conducted on 651 MAGs capable of macromolecule degradation, based on TPM-normalized expression levels of electron acceptor–related genes. **(a)** NMDS ordination shows a significant separation between bottom (n=22) and slope (n=20) samples, indicating distinct transcriptional activity patterns (PERMANOVA, Adonis test; p = 0.043). **(b)** Pielou's evenness index (J′) of electron acceptor–related pathways, showing significantly higher evenness in bottom (n=22) samples compared with slope (n=20) samples (one-sided, wilcox test, **** $p < 0.001$).

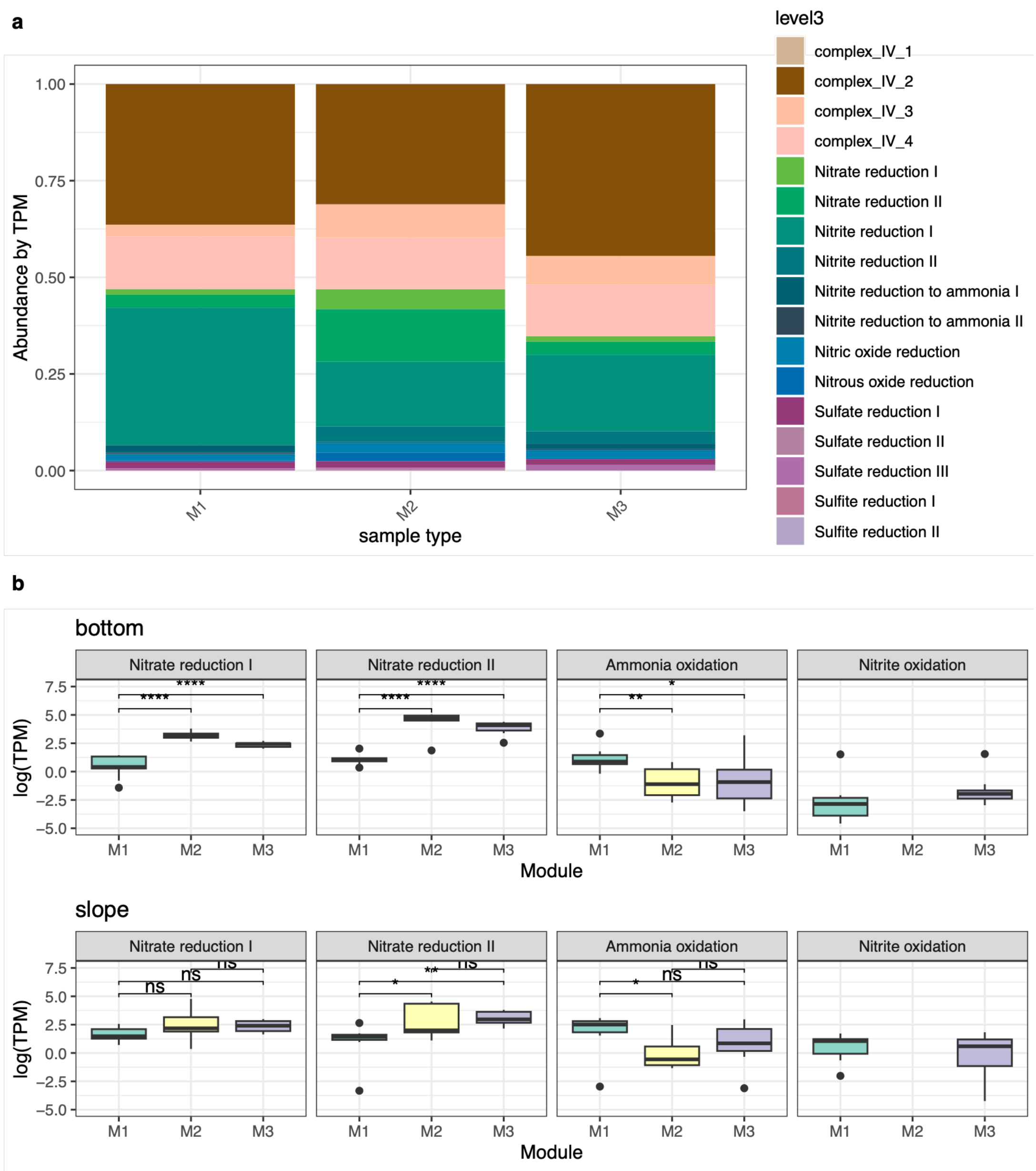

Supplementary Fig. 9

Transcriptional composition of electron acceptors and their sources across co-occurrence network modules. Analyses were performed on three co-occurrence network modules (M1–M3). **(a)** Relative transcriptional composition of electron acceptor–related pathways based on TPM-normalized metatranscriptomic data. **(b)** TPM-normalized transcriptional activity of nitrate- and nitrite-related processes across modules. The upper panels represent bottom samples, and the lower panels represent slope samples. Differences among modules were assessed using the one-sided wilcox

test. Significance levels are indicated as follows: ns, not significant; $*p < 0.05$; $**p < 0.01$; $***p < 0.001$; $****p < 0.0001$.

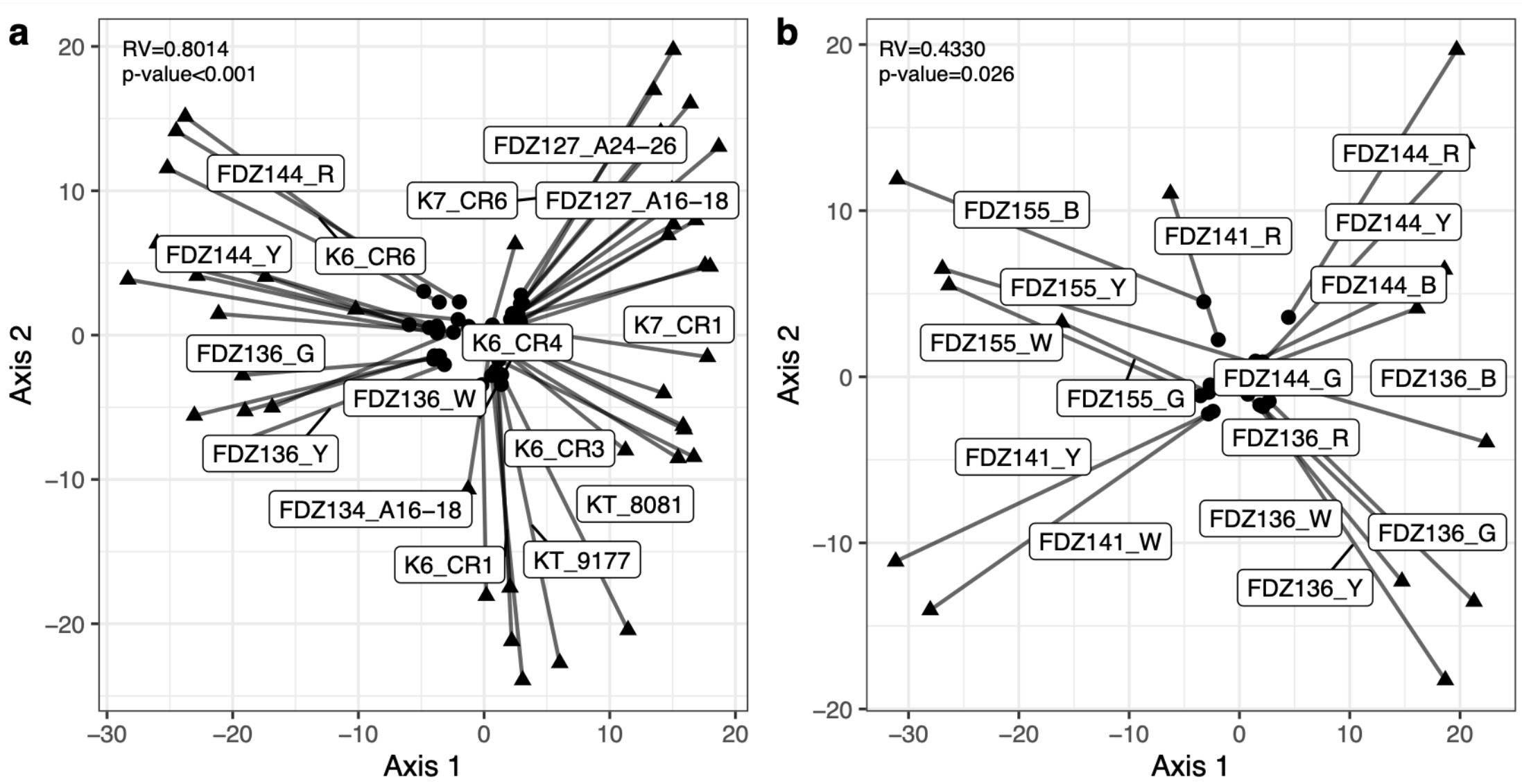

Supplementary Fig. 10

Co-inertia analysis linking electron acceptor–related genes and MAG taxonomic composition. Co-inertia analyses were performed to evaluate the covariance between electron acceptor–related gene profiles and the taxonomic composition of MAGs. **(a)** Co-inertia analysis between TPM-normalized abundances of electron acceptor–related genes derived from metagenomic data and MAG taxonomic composition (n = 42). **(b)** Co-inertia analysis between TPM-normalized expression levels of electron acceptor–related genes derived from metatranscriptomic data and the taxonomic composition of MAGs in the corresponding samples (n = 16). Arrows indicate the joint structure between the two datasets for each sample. The strength and significance of the co-structure are quantified by the RV coefficient and associated permutation test P values.

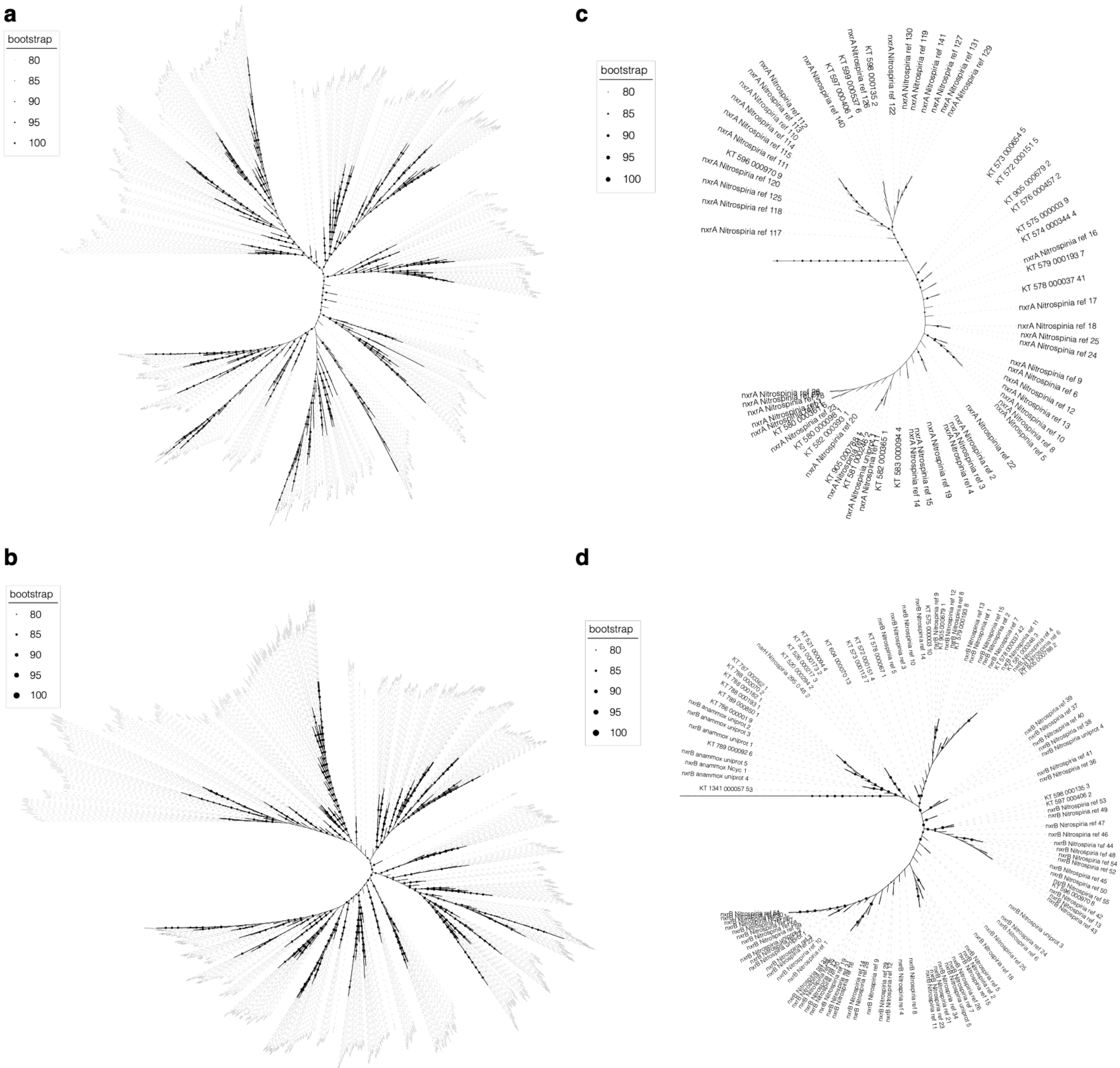

Supplementary Fig. 11

Phylogenetic resolution of nxrA/nxrB and narG/narH homologues within K00370 and K00371. Phylogenetic tree of genes assigned to **(a)** K00370, and **(b)** K00371. **(c)** Pruned clade extracted from panel (a), highlighting sequences annotated as *nxrA*. **(d)** Pruned clade extracted from panel (b), highlighting sequences annotated as *nxrB*. All remaining sequences are annotated as *narG* or *narH* genes. Branch length was ignored.

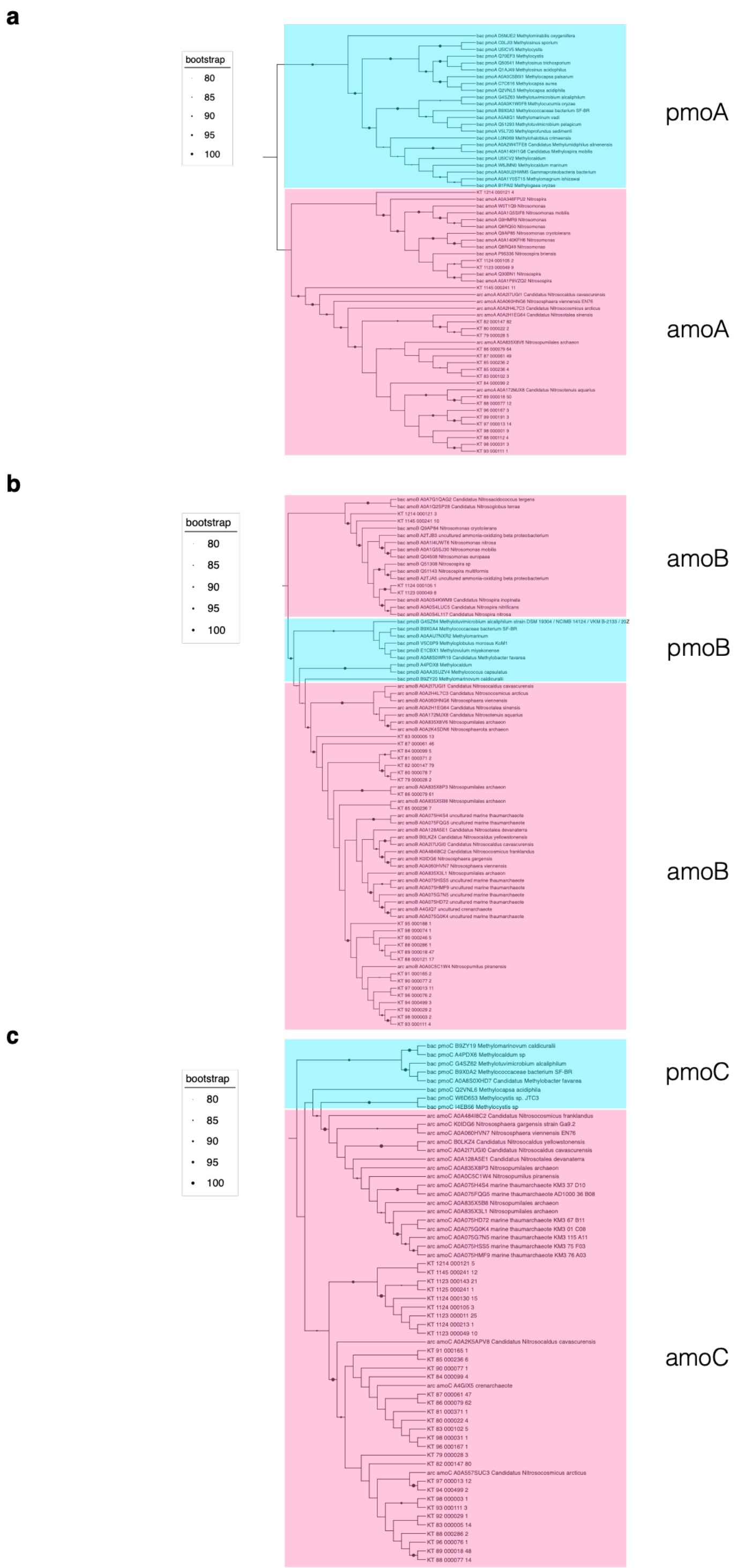

Supplementary Fig. 12

Phylogenetic relationships among genes assigned to K10944, K10945, and K10946. Phylogenetic tree of genes assigned to **(a)** K10944, **(b)**

K10945, and **(c)** K10946. Genes with different functions are distinguished by different colored blocks: blue indicates genes associated with methane oxidation, whereas pink indicates genes associated with ammonia oxidation. Branch length was ignored.

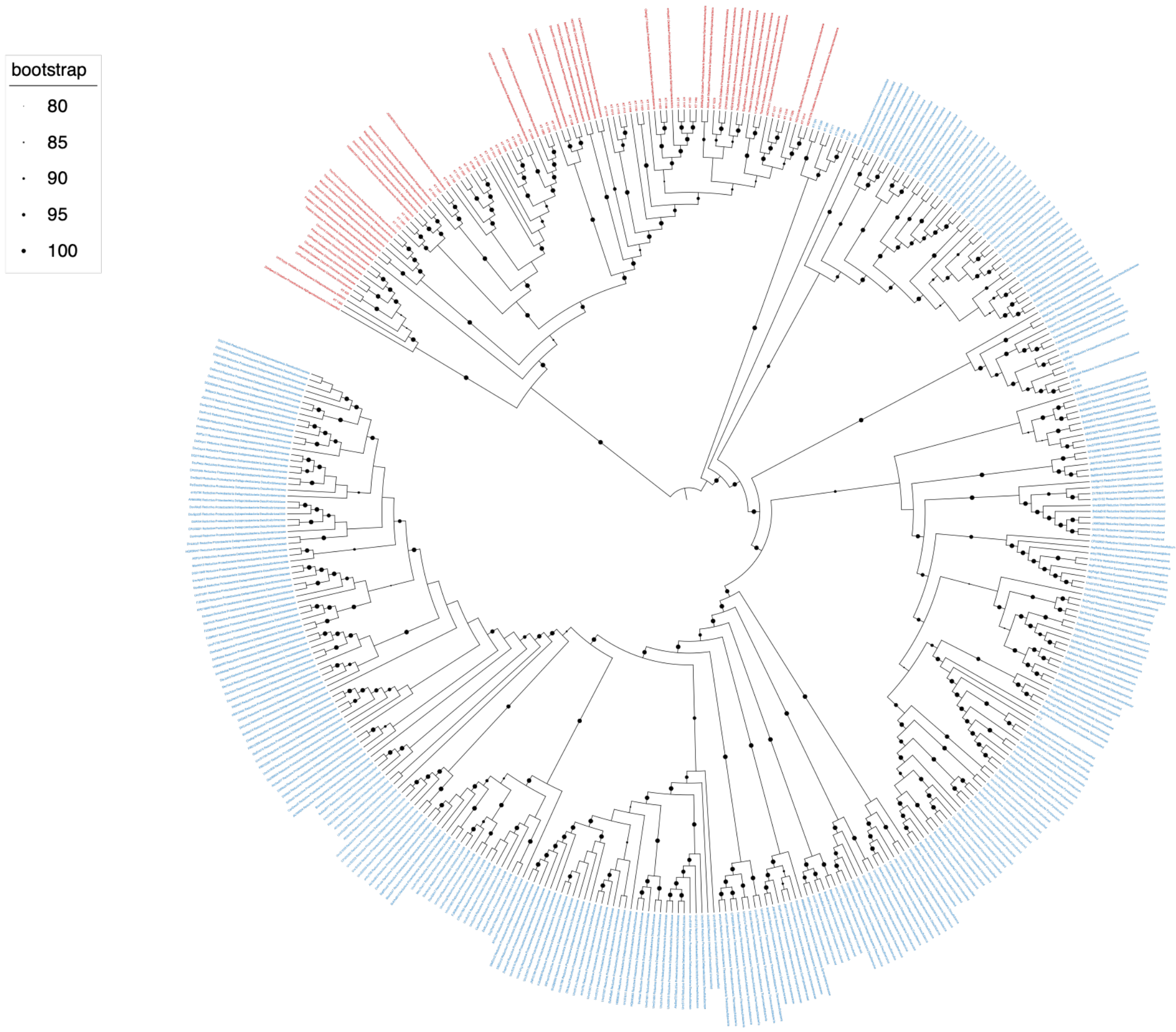

Supplementary Fig. 13

Phylogenetic relationships of *dsrAB* genes from MAGs and reference sequences. Genes with different functions are distinguished by different colors: blue indicates reductive *dsrAB*, whereas red indicates oxidative *dsrAB*. Branch length was ignored.

References

1. Chen, S. Ultrafast one-pass FASTQ data preprocessing, quality control, and deduplication using fastp. *iMeta* **2**, e107 (2023).

2. Li, D. *et al.* MEGAHIT v1.0: A fast and scalable metagenome assembler driven by advanced methodologies and community practices. *Pan-omics analysis of biological data* **102**, 3–11 (2016).

3. Langmead, B. & Salzberg, S. L. Fast gapped-read alignment with bowtie 2. *Nat. Methods* **9**, 357–359 (2012).

4. Wang, Z. *et al.* Effective binning of metagenomic contigs using contrastive multi-view representation learning. *Nat Commun* **15**, 585 (2024).

5. Pan, S., Zhao, X.-M. & Coelho, L. P. SemiBin2: self-supervised contrastive learning leads to better MAGs for short- and long-read sequencing. *Bioinformatics* **39**, i21–i29 (2023).

6. Wang, Z., Huang, P., You, R., Sun, F. & Zhu, S. MetaBinner: a high-performance and stand-alone ensemble binning method to recover individual genomes from complex microbial communities. *Genome Biol* **24**, 1 (2023).

7. Mainguy, J. & Hoede, C. Binette: a fast and accurate bin refinement tool to construct high quality metagenome assembled genomes. *J. Open Source Software* **9**, 6782 (2024).

8. Parks, D. H., Imelfort, M., Skennerton, C. T., Hugenholtz, P. & Tyson, G. W. CheckM: assessing the quality of microbial genomes recovered from isolates, single cells, and metagenomes. *Genome Res.* **25**, 1043–1055 (2015).

9. Chaumeil, P.-A., Mussig, A. J., Hugenholtz, P. & Parks, D. H. GTDB-Tk v2: memory friendly classification with the genome taxonomy database. *Bioinformatics* **38**, 5315–5316 (2022).

10. Parks, D. H. *et al.* GTDB: an ongoing census of bacterial and archaeal diversity through a phylogenetically consistent, rank normalized and

complete genome-based taxonomy. *Nucleic Acids Res.* **50**, gkab776- (2021).

11. Olm, M. R., Brown, C. T., Brooks, B. & Banfield, J. F. dRep: a tool for fast and accurate genomic comparisons that enables improved genome recovery from metagenomes through de-replication. *ISME J* **11**, 2864–2868 (2017).
12. Shaw, J. & Yu, Y. W. Fast and robust metagenomic sequence comparison through sparse chaining with skani. *Nat Methods* **20**, 1661–1665 (2023).
13. Hyatt, D. *et al.* Prodigal: prokaryotic gene recognition and translation initiation site identification. *BMC Bioinform.* **11**, 119 (2010).
14. Aramaki, T. *et al.* KofamKOALA: KEGG Ortholog assignment based on profile HMM and adaptive score threshold. *Bioinformatics* **36**, 2251–2252 (2020).
15. Cantalapiedra, C. P., Hernández-Plaza, A., Letunic, I., Bork, P. & Huerta-Cepas, J. eggNOG-mapper v2: Functional Annotation, Orthology Assignments, and Domain Prediction at the Metagenomic Scale. *Molecular Biology and Evolution* **38**, 5825–5829 (2021).
16. Buchfink, B., Reuter, K. & Drost, H.-G. Sensitive protein alignments at tree-of-life scale using DIAMOND. *Nat Methods* **18**, 366–368 (2021).
17. The UniProt Consortium *et al.* UniProt: the Universal Protein Knowledgebase in 2023. *Nucleic Acids Research* **51**, D523–D531 (2023).
18. Huerta-Cepas, J. *et al.* eggNOG 5.0: a hierarchical, functionally and phylogenetically annotated orthology resource based on 5090 organisms and 2502 viruses. *Nucleic Acids Research* **47**, D309–D314 (2019).
19. Lv, Y., Yang, S., Xiao, X. & Zhang, Y. Stimulated Organic Carbon Cycling and Microbial Community Shift Driven by a Simulated Cold-Seep Eruption. *mBio* **13**, e00087-22 (2022).
20. Zheng, J. *et al.* dbCAN3: automated carbohydrate-active enzyme and substrate annotation. *Nucleic Acids Research* **51**, W115–W121 (2023).
21. Li, W. *et al.* Benchmarking and optimizing qualitative and quantitative

pipelines in environmental metatranscriptomics using mixture controlling experiments. *ISME Communications* **5**, ycaf090 (2025).

22. Deng, Z.-L., Münch, P. C., Mreches, R. & McHardy, A. C. Rapid and accurate identification of ribosomal RNA sequences via deep learning. *Nucleic Acids Res.* **50**, e60–e60 (2022).

23. Liao, Y., Smyth, G. K. & Shi, W. featureCounts: an efficient general purpose program for assigning sequence reads to genomic features. *Bioinformatics* **30**, 923–930 (2014).

24. Yang, N., Lv, Y., Ji, M., Wu, S. & Zhang, Y. High hydrostatic pressure stimulates microbial nitrate reduction in hadal trench sediments under oxic conditions. *Nat Commun* **15**, 2473 (2024).

25. Yamada, K. D., Tomii, K. & Katoh, K. Application of the MAFFT sequence alignment program to large data—reexamination of the usefulness of chained guide trees. *Bioinformatics* **32**, 3246–3251 (2016).

26. Capella-Gutiérrez, S., Silla-Martínez, J. M. & Gabaldón, T. trimAl: a tool for automated alignment trimming in large-scale phylogenetic analyses. *Bioinformatics* **25**, 1972–1973 (2009).

27. Minh, B. Q. *et al.* IQ-TREE 2: New models and efficient methods for phylogenetic inference in the genomic era. *Mol. Biol. Evol.* **37**, 1530–1534 (2020).

28. Letunic, I. & Bork, P. Interactive tree of life (iTOL) v6: Recent updates to the phylogenetic tree display and annotation tool. *Nucleic Acids Res.* **52**, W78–W82 (2024).
