# Supplementary material for: Redox-stratified macromolecule degradation supports microbial survival in the oligotrophic Kermadec Trench sediments
Source: Nat Commun. 2026 Aug 25;17:10138. doi: 10.1038/s41467-026-77249-x (PMC13601601; doi:10.1038/s41467-026-77249-x)
Supplement: Supplementary file 2 [file 41467_2026_77249_MOESM2_ESM.pdf]

Description of Additional Supplementary Files

File Name: Supplementary Data 1
Description: Detailed information on the metagenomic and metatranscriptomic datasets analyzed in this study, including sample ID, sample site, location, water depth (mbsl), latitude, longitude, sediment depth (cmbsf), accession numbers, and data source.

File Name: Supplementary Data 2
Description: Detailed genomic and taxonomic information for the metagenome-assembled genomes (MAGs) reconstructed in this study, including MAG ID, completeness, contamination, and taxonomic assignment.

File Name: Supplementary Datab3
Description: Detailed information on the top 10 most actively transcribed glycoside hydrolases (GHs), including their GH-family classifications, predicted substrate group, predicted substrate, predicted product, and title of reference.

File Name: Supplementary Data 4
Description: Predicted metabolic functions of the reconstructed MAGs inferred from their actively transcribed genes.

File Name: Supplementary Data 5
Description: A comprehensive list of the KEGG Orthology (KO) identifiers included in this study, together with their functional categories.

File Name: Supplementary Data 6
Description: Detailed information and supporting data for each phylogenetic tree constructed in this study, including corresponding figures, model, remained columns, and sequence number.
