# Supplementary material for: Redox-stratified macromolecule degradation supports microbial survival in the oligotrophic Kermadec Trench sediments
Source: Nat Commun. 2026 Aug 25;17:10138. doi: 10.1038/s41467-026-77249-x (PMC13601601; doi:10.1038/s41467-026-77249-x)
Supplement: Supplementary file 3 [file 41467_2026_77249_MOESM4_ESM.pdf]

nature portfolio

Peer Review File

Redox-stratified macromolecule degradation supports microbial survival in the oligotrophic Kermadec Trench sediments

Corresponding Author: Professor Yu Zhang

Parts of this Peer Review File have been redacted as indicated to maintain confidentiality.

This file contains all reviewer reports in order by version, followed by all author rebuttals in order by version.

Version 0:

Reviewer comments:

Reviewer #1

(Remarks to the Author)
I was a reviewer on a related manuscript [REDACTED]. I was asked by an editor at Nature Communications to also review this manuscript (at a high-level rather than in detail) and to provide feedback on whether I think it would make sense for the manuscripts to be combined.

Regarding the overlap in the two articles: I think the focuses are sufficiently separate that it makes sense for them to be separate articles. However, it would be important to clearly cite each other and not report duplicate results or methods without making it clear this was part of a larger project with multiple studies.

The samples collected from the hadal zone are clearly precious, and I think publishing these data and basic descriptive data regarding them would be valuable for the field. The observations of magnetotactic bacteria and high proportion of heterotrophic MAGs is interesting, although I lack the background to evaluate the specific genes involved and the novelty of the claims.

I am skeptical about some of the authors' claims regarding these data. In particular, much of their work is based on co-occurrence networks based on 9 samples. This is an extremely small dataset and one shouldn't generalize about associations between variables from such a sampling, at least not for microbiome data, which is extremely noisy and requires high sample sizes to ensure robust results. The networks are also based on Pearson correlation coefficients, which are inappropriate for these datatypes as they do not fulfill the assumptions of this approach (compositional data requires more specialized methods, and Pearson is highly sensitive to outliers). I believe the Mantel test approach is more reasonable given that it's focused on the presence of genes across genomes, if I am understanding correctly. If this approach is also based on comparing genes across samples then this would face the same limitation.

Much like the other manuscript, the limitations of this study and impact on the interpretations should also be outlined very clearly. The authors present their model in a definitive way, but this is a new hypothesized model rather than confirmation of a model. This is clearly seen in the conclusion where unambiguous causative language is used, such as "MTBs contribute to the vertical transport of…". There is no direct evidence of this and other results (except for cases where you do have follow-up experiments confirming MTB bacteria are indeed present for instance), and this framing of their results is inappropriate and misleading. Instead, it would be appropriate to state what you have observed (the presence of these bacteria) and then state that you hypothesize they are playing a role of vertical transmission for nutrients. The same is true for the other causal mechanisms hypothesized in this manuscript.

Minor
The methods start with "For 9 sediment samples were collected from the Mariana Trench
during cruises TS29 in 2022". Just flagging this to be changed to Kermadec.

I noticed numerous grammatical errors to address as well – I suggest the authors use a large language model to aid with

copy editing, if that is permitted by the journal.

Reviewer #2

(Remarks to the Author)
In this manuscript, the authors investigate, through MAGs and metatranscriptomes, the metabolic potential of bacteria inhabiting the hadal zone of the Kermadec Trench. Based on my expertise, I find no major issues with the analyses used to identify the genetic determinants involved in macromolecule degradation and terminal electron acceptors. However, I am more critical of the conclusions drawn from the analyses on magnetotactic bacteria (MTB). Below, I outline the points of disagreement I have with this manuscript.

Results section
The first paragraph presents the sequencing results, including the MAGs and metatranscriptomes obtained.
The second paragraph details the repertoire of genes found in the MAGs that are involved in carbohydrate degradation. It is shown here that the vast majority of reconstructed genomes appear to belong to heterotrophic bacteria capable of degrading macromolecules.
The third paragraph explores potential compounds used as terminal electron acceptors, based on both metatranscriptomic and metagenomic data. This section indicates that most MAGs belong to aerobic bacteria, followed by facultative anaerobes, and finally strict anaerobes. The authors further show that bacteria capable of degrading macromolecules seem to mainly use oxygen as the terminal electron acceptor.
The fourth paragraph concerns the identification of MAGs containing magnetosome gene clusters (MGCs) in MTB. Twenty-two MAGs are identified. In addition, the authors report the presence of crystals resembling magnetite, typical of MTB, in some of the samples from which the MAGs were obtained. The species-level diversity of these potential MTB MAGs is noteworthy. The second most represented phylum is Actinomycetota (>15%), yet the authors do not comment on this point, even though this phylum has never previously been reported to contain MTB. This is an important results that should be highlighted.
When reading this section of the results, it is not entirely clear how the paragraph on MTB fits within the overall flow of the manuscript. I believe the structure of the Results section could be revised to improve clarity and logical organization. Simply improving the transition between the third and fourth, or between the fourth and fifth paragraphs of the Results, could make the text more coherent.
In the final paragraph of the Results, the authors perform a correlation analysis between the phylogenetic affiliation of the different MAGs and their metabolic potential. I was quite surprised by the statement: “MTB could facilitate the transportation of nitrite and sulfate from the oxygenated upper layers to the anoxic deeper zones.”
There is currently no evidence that MTB migrate vertically between the different sediment layers in natural environments. Culture-based studies have shown that magnetotactic bacteria tend to remain within their optimal growth zone. Moreover, a bacterium that uses nitrite or sulfate as a terminal electron acceptor will typically consume these compounds for its own metabolism. It is therefore unclear why an MTB would take up nitrite or sulfate from an oxic zone only to release it into an anoxic one. Since this point is important to the manuscript’s conclusions, it would be helpful to clarify the spatial scale involved: what is the diffusion depth of oxygen in the sampled sediments? Are we talking about micrometer-, millimeter-, or centimeter-scale migration?

Discussion section
Line 357, to my knowledge, there is no evidence that volcanic environments are particularly favorable to the development of MTB. The three references cited do not seem to support this statement either. Because iron is not typically a limiting factor in most aquatic environments, especially in sediments, high iron concentrations alone do not appear to promote the development of a greater number of MTB. Furthermore, culture experiments indicate that low concentrations of soluble iron (around 1 µM) are sufficient for magnetosome formation in MTB.
Line 370, regarding the term “redistributes”, it cannot be assumed that a bacterium would release nutrients essential to its own growth. Is there any evidence supporting this claim?

Reviewer #3

(Remarks to the Author)
Review of “Versatile redox-coupling mechanisms assisting the microbial adaptation in the Kermadec Trench sediment”

This manuscript investigates how microbial communities thrive in the extreme environment of the Kermadec Trench (hadal depth ~10 km). The authors combined in situ RNA fixation during deep-sea submersible dives with metagenomics and meta-transcriptomics from nine sediment samples to capture near-in situ microbial gene expression. They reconstructed 300 metagenome-assembled genomes (MAGs) and identified active microbial degradation of complex macromolecules (e.g. peptidoglycan, polysaccharides) as a key strategy for energy and carbon acquisition. Their data suggest that these degradation processes are coupled with both oxygen and alternative electron acceptors (like nitrite and sulfate). Notably, the study presents evidence for magnetotactic bacteria (MTB) in hadal sediments, confirmed by transmission electron microscopy (TEM) images of magnetite-containing cells and rock magnetic measurements, and proposes that MTB vertically migrate to shuttle oxidants (nitrite, sulfate) from oxic surface layers to deeper anoxic zones. This “redox-coupling” mechanism is invoked to explain the paradox of unexpectedly high microbial activity in such an extreme, oligotrophic environment.

Overall, the manuscript is interesting, and its novelty lies in its multiple approach (in situ preservation, multi-omics, microscopy, geophysics) and a conceptual model of versatile redox coupling that enhances survival under high pressure.

The sampling strategy is appropriate. Sediment cores (0–10 cm depth) were collected from hadal depths using the Fendouzhe manned submersible. In situ RNA fixation was performed immediately on the seafloor to preserve the native transcriptional state. The use of in situ RNA fixation directly addresses the concern of decompression artifacts. The authors recognize this.

Some Potential Weaknesses remain, namely RNAlater needs to infiltrate sediment quickly to halt cellular activity. The manuscript does not detail how the preservative was applied (e.g. injected into cores or sub-samples taken and dropped into RNAlater vials). If whole 0–10 cm cores were simply placed in RNAlater, one might worry whether the fixative permeated interior sediments rapidly enough to prevent any post-sampling shifts. The authors need to specify in detail the procedure used, otherwise the ms lacks replicability.

Pressure changes might have occurred during fixation? It's implied that samples were fixed at ambient pressure on the seafloor, but if the RNAlater container was opened at depth and then sealed, the sample still undergoes decompression on ascent. These aspects require clarification.

The study generated a comprehensive multi-omics dataset from the hadal sediment samples. DNA and RNA were co-extracted from the preserved samples and high-throughput sequenced. The authors performed quality control and co-assembly of reads prior to binning. They then reconstructed MAGs and focused subsequent analyses on high-quality, non-redundant MAGs meeting thresholds of >50% completeness and <5% contamination. Metatranscriptomic reads (after rRNA depletion) were mapped back to a comprehensive gene catalog to quantify gene expression levels. This approach is robust and ensures that even genes from organisms not fully captured in MAG bins are counted in expression analyses.

Overall, the laboratory and computational procedures for obtaining and processing the omics data are complete and state-of-the-art, consistent with previously published deep-sea metagenomic studies. The data quality appears sufficient to support the conclusions.

Yet, the authors retained genomes with >50% completeness (and <5% contamination). By conventional standards >50% would be considered medium-quality draft genomes rather than high-quality. Many of the MAGs used for functional analysis could be missing nearly half their genes. This raises a caution: when they report, for example, that "71 MAGs were incapable of generating required electron acceptors (lacking nitrite or sulfate-producing pathways)", some MAGs might appear to lack a pathway simply because that part of the genome wasn't recovered. If a MAG is 60% complete, the absence of a nitrification gene could be due to incomplete assembly rather than true absence in the living organism.

It would strengthen confidence if the authors indicate how many of those 71 MAGs were near-complete vs. just above 50%, or if they performed any cross-check (for example, searching unbinned contigs for those functions). Additionally, co-assembling all samples could, in theory, create chimeric bins if closely related strains differ between sites. The manuscript doesn't detail the binning method or any manual curation steps. This is a standard practice but requires in depth explanation.

The downstream bioinformatic analyses in the manuscript are extensive and generally appropriate. The authors leveraged their annotated MAGs and gene expression data to infer metabolic capacities and active pathways in the community.

However, a challenge is the limited sample size (n=9 sediment samples). The authors set a relatively high Pearson r threshold (≥0.6) for edges in the network, which is a reasonable filter, but significance testing with n=9 has low power. There is a risk of both false positives and false negatives in the network: some observed correlations might be spurious (driven by unmeasured factors or the compositional nature of relative abundance data), while some real interactions might not register due to the small sample size. The authors did mitigate this by requiring that MAGs appear in ≥90% of samples to be included. Yet, the interpretation should be softened and presented as hypothesis-generating rather than as a proof. Correlation does not equal causation. A brief note on how they normalized abundance data would be also useful.

There are few weaknesses also on electron microscope analyses. One minor concern is the abundance and significance of MTB: magnetotactic bacteria constitute a relatively small fraction of the community. The TEM images come from a concentrated search or enrichment. It's not stated how many cells or fields of view were examined to find the magnetosome-containing bacteria. If only a handful of cells were observed, one could question how representative or influential they are in situ.

The impact of artifacts for this specific studied could be limited, but an explanation on how they handled or at least acknowledge this issue would be appropriate in a revision. The authors infer cross-feeding of electron acceptors based on absence/presence of genes, but they have not directly carried out measurements. The manuscript would benefit from explicit acknowledgment of this and perhaps suggestions for future experimental validation.

A central question motivating this work is why microbial biomass and activity in the trench are higher than expected for such an extreme, oligotrophic environment. The authors propose a compelling mechanistic interpretation: extreme pressure itself drives microbes to adopt versatile redox strategies that sustain high metabolic activity despite low oxygen.

The mechanistic story is largely consistent with the data. The authors' multi-omic evidence clearly demonstrates diverse

metabolic capabilities in the community: genes for aerobic respiration and anaerobic respiration were found actively expressed. The authors rightly highlight that MTB have been overlooked in deep-sea sediments and raise the possibility that MTB are integral to element cycling in trenches.

The main caveat is that much of this remains inference without direct experimentation. For example, the manuscript assumes that because transcripts were captured in situ, the high metabolic gene expression is truly happening at depth. However, could any of the "high activity" signals still be influenced by the sample handling? If RNAlater did not instantly fixed? The authors did not detect or mention any obvious artifacts.

Another uncertainty is how universal or large the MTB effect is. With MTB-related reads being a few percent of the community, can we be sure their vertical shuttling substantially fuels the rest of the microbes? The co-occurrence links imply many degraders could benefit from MTB, but it's not direct proof.

The authors' interpretations would also be strengthened by acknowledging alternative explanations for high activity: for instance, simply the higher input of organic matter (as noted) and possibly physiological adaptations like piezolytes or specialized enzymes could contribute to resilience under pressure. The focus on redox coupling is interesting, but a broader context of hadal adaptation (e.g. pressure-resistant enzyme structures, membrane adaptations, etc.) is beyond this study's scope and is rightly left to citations.

Some key conclusions (e.g., metabolic cooperation between organisms, the extent of MTB's role) are based on correlation and association rather than direct measurement. This is a natural limitation of environmental 'omics studies, but it means causality is inferred, not proven. A reader should keep in mind that alternative interpretations of the correlations are possible (though the authors' interpretations are well-reasoned).

IUsing MAGs with as low as 50% completeness could omit genes, so any conclusions that certain pathways are absent should be cautious. There is uncertainty in declaring what a microbe "cannot do" when its genome is partial, better to stick on what they can do.

Small errors can be easily addressed (such as referring to "Mariana Trench" instead of Kermadec in the Methods).

In conclusion, this manuscript presents sound, well-supported findings and employs a robust, cutting-edge methodological framework suitable for publication after appropriate revision. The core conclusions, that hadal trench microbes use versatile redox-coupling mechanisms (including possible magnetotactic shuttling) to maintain high metabolic activity under extreme pressure, are both novel and important for the field of deep-sea microbial ecology.

Version 1:

Reviewer comments:

Reviewer #1

(Remarks to the Author)
The authors have done a good job addressing my comments overall.

However, there is still insufficient discussion of the limitations of their study. They mention in their response that "We now explicitly outline the limitations of genome completeness, sample size, and the inference-based nature of our metabolic conclusions (Lines 379-382)." I do not see that actually added into their manuscript, and lines 379-382 do not match that claim. This needs to be added clearly and extensively. A single sentence would be insufficient: a proper discussion of the limitations is needed.

Using publicly available data is valid, but the authors should also consider to what degree technical differences between the samples from different sources could be driving the results. This should be explicitly explored when evaluating the key findings. E.g., is there a different proportion of samples from different studies in the slope vs. bottom samples? This would mean your signal was confounded with methodology if so, and should definitely be clearly shown one way or another.

Also note the typo "quatatum" on line 382.

Reviewer #2

(Remarks to the Author)
I thank the authors for addressing my main comment regarding the magnetotactic bacteria and for restructuring the Results section accordingly. I have no further comments on this manuscript within my area of expertise.

Reviewer #4

(Remarks to the Author)
I was asked to participate in this review process as a mediator, following unavailability of reviewer 3 for a review of the

revised manuscript. As such, I have only considered whether the authors' responses clearly address the reviewer's comments.

Overall I would say this is the case, with the authors taking pains to increase the statistical power of their analysis, clarifying methods and removing a debated section. A few minor problems remain in my opinion:
- L379-382: The authors claim that this sentence "explicitly outline the limitations of genome completeness, sample size, and the inference-based nature of our metabolic conclusions" and highlights "the constraints of their approach and their implications for data interpretation", which does not appear to be the case. The sentence also seems to not make sense. I would suggest rephrasing and properly discussing the limitations of omics approaches without accompanying geochemistry data instead of simply suggesting avenues for further studies.
- L385-390: In the response to the reviewer, the authors state that RNAlater was injected into the sediment cores before sealing them in situ. On the other hand the manuscript states "Surface sediments (0 ~ 10 cmbsf) were collected and immersed in RNAlater in situ" which is contradictory. Please fix this discrepancy and precisely report how the samples were collected, the amount of RNAlater that was injected or used for immersion, etc…
- L393: The correct citation for 51 (metagenomes produced from expedition TAN1711 to the Kermadec trench) should be Trouche et al., 2023 (https://doi.org/10.1038/s43705-023-00341-6). Although the current citation (Schauberger et al., 2024) analyses data deposited in the same ENA project, it is Trouche et al. that describes the metagenomes and metadata used here.

Despite these minor remaining points, the authors have produced a rigorous and interesting study that will be a valuable contribution to our understanding of microbial life in hadal sediments.

Open Access This Peer Review File is licensed under a Creative Commons Attribution 4.0 International License, which permits use, sharing, adaptation, distribution and reproduction in any medium or format, as long as you give appropriate credit to the original author(s) and the source, provide a link to the Creative Commons license, and indicate if changes were made.
In cases where reviewers are anonymous, credit should be given to 'Anonymous Referee' and the source.
The images or other third party material in this Peer Review File are included in the article's Creative Commons license, unless indicated otherwise in a credit line to the material. If material is not included in the article's Creative Commons license and your intended use is not permitted by statutory regulation or exceeds the permitted use, you will need to obtain permission directly from the copyright holder.
To view a copy of this license, visit https://creativecommons.org/licenses/by/4.0/

Responses to all reviewers:

We sincerely thank all reviewers for your thoughtful and constructive comments, which significantly improved the clarity and scientific rigor of our manuscript. We have carefully revised the manuscript in response to each point raised. Below, we summarize the major revisions and the core findings of the updated version.

Major Revisions:

1. **Expanded Omics Dataset:**

   We have significantly increased our dataset to enhance statistical robustness. The revised study now includes 42 metagenomic and 16 metatranscriptomic samples from the Kermadec Trench, greatly expanding the scope and representativeness of the analyses.

2. **Improved Methodology and Descriptions:**

   Based on reviewer feedback, we optimized our bioinformatic pipelines, especially for network construction. We now use Spearman correlation and Random Matrix Theory to infer microbial co-occurrence networks, which are more appropriate for microbiome data. Additionally, we have provided a more detailed description of sample collection, in situ RNA fixation, and data processing procedures to ensure reproducibility.

3. **Removal of Speculative MTB Content:**

   All discussions and analyses related to magnetotactic bacteria (MTB)

have been removed to avoid overinterpretation and speculative conclusions, as there was no direct evidence supporting their hypothesized ecological role in this study.

4. **Discussion of Study Limitations:**

   We now explicitly outline the limitations of genome completeness, sample size, and the inference-based nature of our metabolic conclusions (Lines 379-382).

Core Scientific Findings of the Revised Manuscript:

1. **Redox-Coupled Macromolecule Degradation:**

   We show that nearly half of the microbial MAGs in Kermadec Trench sediments actively transcribe genes for macromolecule degradation and simultaneously express pathways for utilizing non-oxygen terminal electron acceptors (e.g., nitrate, nitrite, sulfate). This metabolic versatility allows microbial communities to survive in the oligotrophic and low-oxygen conditions of the hadal zone.

2. **Environmental Stratification and Community Differentiation:**

   The trench’s V-shaped topography results in uneven POM accumulation, which contributes to redox stratification between slope (6,000–9,000 m) and bottom (>9,000 m) habitats. This drives divergence in microbial community composition and metabolic strategies:

- Slope communities show higher expression of genes associated with strong oxidants like oxygen and nitrite.
- Bottom communities utilize weaker oxidants (e.g., N_2O) but maintain similar macromolecule degradation activity, reflecting adaptation to more reductive environments.

These findings illustrate how electron acceptor flexibility and functional convergence in macromolecule recycling underpin microbial persistence in one of Earth's most nutrient-limited environments. Once again, we thank the reviewers for their time and valuable feedback. We hope that the revised manuscript now meets the expectations and scientific standards for publication. We have also made detailed revisions and responses to each of your comments, as outlined below.

Reviewers' comments:

Reviewer #1 (Remarks to the Author):

I was a reviewer on a related manuscript **[REDACTED]**. I was asked by an editor at Nature Communications to also review this manuscript (at a high-level rather than in detail) and to provide feedback on whether I think it would make sense for the manuscripts to be combined.

Regarding the overlap in the two articles: I think the focuses are sufficiently separate that it makes sense for them to be separate articles. However, it would be important to clearly cite each other and not report duplicate results or methods without making it clear this was part of a larger project with multiple studies.

The samples collected from the hadal zone are clearly precious, and I think publishing these data and basic descriptive data regarding them would be valuable for the field. The observations of magnetotactic bacteria and high proportion of heterotrophic MAGs is interesting, although I lack the background to evaluate the specific genes involved and the novelty of the claims.

I am skeptical about some of the authors' claims regarding these data. In particular, much of their work is based on co-occurrence networks based on 9 samples. This is an extremely small dataset and one shouldn't generalize about associations between variables from such a sampling, at least not for microbiome data, which is extremely noisy and requires high sample sizes to ensure robust results. The networks are also based on Pearson correlation coefficients, which are inappropriate for these datatypes as they do not fulfill the assumptions of this approach (compositional data requires more specialized methods, and Pearson is

highly sensitive to outliers). I believe the Mantel test approach is more reasonable given that it's focused on the presence of genes across genomes, if I am understanding correctly. If this approach is also based on comparing genes across samples then this would face the same limitation.

Response: Thanks for your valuable comments. (1) We fully acknowledge the limitation of the small sample size used in the previous version of our manuscript. To address this concern, we have now substantially expanded our dataset. Specifically, we incorporated publicly available metagenomic data from hadal sediment samples in the Kermadec Trench, and we obtained newly sequenced data from additional samples provided by our new collaborator, Dr. Huahua Jian. As a result, our study now includes 42 metagenomic and 16 metatranscriptomic samples. This number of metagenomic datasets is sufficient for constructing a robust microbial co-occurrence network[1,2]. (2) Furthermore, in response to the reviewer's concern regarding the use of Pearson correlation for co-occurrence analysis, we have revised our methodology accordingly. We now use Spearman correlation to construct co-occurrence networks, compute correlation matrices, and perform the Mantel tests. Spearman correlation is more appropriate for sparse microbiome data and is less sensitive to outliers and compositional effects than Pearson correlation. All related sections have been updated.

Ref.

1. Agyapong, D., Propster, J. R., Marks, J. & Hocking, T. D. Cross-validation for training and testing co-occurrence network inference algorithms. BMC Bioinf. 26, 74 (2025).

2. Kishore, D. et al. Inferring microbial co-occurrence networks from amplicon data: a systematic evaluation. mSystems e00961-22 (2023) doi:10.1128/msystems.00961-22.

Much like the other manuscript, the limitations of this study and impact on the interpretations should also be outlined very clearly. The authors present their model in a definitive way, but this is a new hypothesized model rather than confirmation of a model. This is clearly seen in the conclusion where unambiguous causative language is used, such as "MTBs contribute to the vertical transport of…". There is no direct evidence of this and other results (except for cases where you do have follow-up experiments confirming MTB bacteria are indeed present for instance), and this framing of their results is inappropriate and misleading. Instead, it would be appropriate to state what you have observed (the presence of these bacteria) and then state that you hypothesize they are playing a role of vertical transmission for nutrients. The same is true for the other causal mechanisms hypothesized in this manuscript.

Response: Thanks for your valuable suggestions. We agree that, in its original form, parts of the manuscript overstated causal interpretations and

did not sufficiently emphasize the limitations of the study or the hypothetical nature of the proposed model. 1) Regarding the role of magnetotactic bacteria (MTB): We agree that, in the present study, there is no direct experimental evidence demonstrating that MTB actively contribute to vertical transport processes. As another reviewer raised a similar concern, we have decided to remove the MTB-related discussion entirely from the revised manuscript to avoid speculative or potentially misleading interpretations. This revision ensures that all remaining interpretations are directly supported by the data presented in this study. 2) Regarding causal language and framing of conclusions: We now describe our findings in terms of the observed presence and distribution patterns of microorganisms, and explicitly frame any proposed mechanisms as hypotheses rather than confirmed processes. Definitive or unambiguous causative language has been removed throughout the manuscript to ensure a more cautious and appropriate interpretation of the results. In addition, we have substantially strengthened the discussion of study limitations. The constraints of our approach and their implications for data interpretation are now explicitly outlined in Lines 379–382 of the revised manuscript. These revisions clarify that the proposed model is a hypothesized framework based on observed patterns, rather than a confirmed causal model.

Minor

The methods start with “For 9 sediment samples were collected from the Mariana Trench during cruises TS29 in 2022”. Just flagging this to be changed to Kermadec.

Response: We have corrected in Line 385. Thanks!

I noticed numerous grammatical errors to address as well – I suggest the authors use a large language model to aid with copy editing, if that is permitted by the journal.

Response: As you suggested, we carefully reviewed and polished the revised manuscript.

Reviewer #2 (Remarks to the Author):

In this manuscript, the authors investigate, through MAGs and metatranscriptomes, the metabolic potential of bacteria inhabiting the hadal zone of the Kermadec Trench. Based on my expertise, I find no major issues with the analyses used to identify the genetic determinants involved in macromolecule degradation and terminal electron acceptors. However, I am more critical of the conclusions drawn from the analyses on magnetotactic bacteria (MTB). Below, I outline the points of

disagreement I have with this manuscript.

Results section

The first paragraph presents the sequencing results, including the MAGs and metatranscriptomes obtained. The second paragraph details the repertoire of genes found in the MAGs that are involved in carbohydrate degradation. It is shown here that the vast majority of reconstructed genomes appear to belong to heterotrophic bacteria capable of degrading macromolecules. The third paragraph explores potential compounds used as terminal electron acceptors, based on both metatranscriptomic and metagenomic data. This section indicates that most MAGs belong to aerobic bacteria, followed by facultative anaerobes, and finally strict anaerobes. The authors further show that bacteria capable of degrading macromolecules seem to mainly use oxygen as the terminal electron acceptor. The fourth paragraph concerns the identification of MAGs containing magnetosome gene clusters (MGCs) in MTB. Twenty-two MAGs are identified. In addition, the authors report the presence of crystals resembling magnetite, typical of MTB, in some of the samples from which the MAGs were obtained. The species-level diversity of these potential MTB MAGs is noteworthy. The second most represented phylum is Actinomycetota (>15%), yet the authors do not comment on this point, even though this phylum has never previously been reported to

contain MTB. This is an important results that should be highlighted. When reading this section of the results, it is not entirely clear how the paragraph on MTB fits within the overall flow of the manuscript. I believe the structure of the Results section could be revised to improve clarity and logical organization. Simply improving the transition between the third and fourth, or between the fourth and fifth paragraphs of the Results, could make the text more coherent.

In the final paragraph of the Results, the authors perform a correlation analysis between the phylogenetic affiliation of the different MAGs and their metabolic potential. I was quite surprised by the statement: “MTB could facilitate the transportation of nitrite and sulfate from the oxygenated upper layers to the anoxic deeper zones.” There is currently no evidence that MTB migrate vertically between the different sediment layers in natural environments. Culture-based studies have shown that magnetotactic bacteria tend to remain within their optimal growth zone. Moreover, a bacterium that uses nitrite or sulfate as a terminal electron acceptor will typically consume these compounds for its own metabolism. It is therefore unclear why an MTB would take up nitrite or sulfate from an oxic zone only to release it into an anoxic one. Since this point is important to the manuscript’s conclusions, it would be helpful to clarify the spatial scale involved: what is the diffusion depth of oxygen in the

sampled sediments? Are we talking about micrometer-, millimeter-, or centimeter-scale migration?

Discussion section

Line 357, to my knowledge, there is no evidence that volcanic environments are particularly favorable to the development of MTB. The three references cited do not seem to support this statement either. Because iron is not typically a limiting factor in most aquatic environments, especially in sediments, high iron concentrations alone do not appear to promote the development of a greater number of MTB. Furthermore, culture experiments indicate that low concentrations of soluble iron (around 1 µM) are sufficient for magnetosome formation in MTB.

Line 370, regarding the term “redistributes”, it cannot be assumed that a bacterium would release nutrients essential to its own growth. Is there any evidence supporting this claim?

Response: We agree with the reviewer that this study does not provide direct evidence supporting vertical migration of magnetotactic bacteria (MTB), nor evidence that MTB transport nutrients between different sediment layers. Given these limitations, and considering that a similar concern was raised by another reviewer, we have removed the MTB-

related analyses and interpretations from the revised manuscript to avoid speculative or potentially misleading conclusions.

We also thank the reviewer for the constructive suggestions regarding the overall structure of the Results section. We appreciate the reviewer's time and effort and look forward to their assessment of the revised version of the manuscript.

Reviewer #3 (Remarks to the Author):

Review of "Versatile redox-coupling mechanisms assisting the microbial adaptation in the Kermadec Trench sediment"

This manuscript investigates how microbial communities thrive in the extreme environment of the Kermadec Trench (hadal depth ~10 km). The authors combined in situ RNA fixation during deep-sea submersible dives with metagenomics and meta-transcriptomics from nine sediment samples to capture near-in situ microbial gene expression. They reconstructed 300 metagenome-assembled genomes (MAGs) and identified active microbial degradation of complex macromolecules (e.g. peptidoglycan, polysaccharides) as a key strategy for energy and carbon acquisition. Their

data suggest that these degradation processes are coupled with both oxygen and alternative electron acceptors (like nitrite and sulfate). Notably, the study presents evidence for magnetotactic bacteria (MTB) in hadal sediments, confirmed by transmission electron microscopy (TEM) images of magnetite-containing cells and rock magnetic measurements, and proposes that MTB vertically migrate to shuttle oxidants (nitrite, sulfate) from oxic surface layers to deeper anoxic zones. This “redox-coupling” mechanism is invoked to explain the paradox of unexpectedly high microbial activity in such an extreme, oligotrophic environment.

Overall, the manuscript is interesting, and its novelty lies in its multiple approach (in situ preservation, multi-omics, microscopy, geophysics) and a conceptual model of versatile redox coupling that enhances survival under high pressure.

The sampling strategy is appropriate. Sediment cores (0–10 cm depth) were collected from hadal depths using the Fendouzhe manned submersible. In situ RNA fixation was performed immediately on the seafloor to preserve the native transcriptional state. The use of in situ RNA fixation directly addresses the concern of decompression artifacts. The authors recognize this.

Some Potential Weaknesses remain, namely RNAlater needs to infiltrate sediment quickly to halt cellular activity. The manuscript does not detail how the preservative was applied (e.g. injected into cores or sub-samples taken and dropped into RNAlater vials). If whole 0–10 cm cores were simply placed in RNAlater, one might worry whether the fixative permeated interior sediments rapidly enough to prevent any post-sampling shifts. The authors need to specify in detail the procedure used, otherwise the ms lacks replicability. Pressure changes might have occurred during fixation? It's implied that samples were fixed at ambient pressure on the seafloor, but if the RNAlater container was opened at depth and then sealed, the sample still undergoes decompression on ascent. These aspects require clarification.

Response: We thank the reviewer for raising these important methodological points. During sampling, RNAlater was directly injected into the sediment cores on the seafloor and the cores were immediately sealed under in situ pressure conditions. Therefore, no pressure change occurred during the fixation process itself. The direct injection ensured rapid contact between the preservative and the sediment, minimizing any post-sampling transcriptional activity. RNAlater rapidly kills microbial cells and stabilizes RNA, thereby preventing mRNA degradation. As a result, subsequent pressure changes experienced during sample recovery to the surface are unlikely to affect the preserved transcriptional profiles.

To improve clarity and reproducibility, we revised description of this fixation procedure to the Methods section (Line 385-390) of the revised manuscript.

The study generated a comprehensive multi-omics dataset from the hadal sediment samples. DNA and RNA were co-extracted from the preserved samples and high-throughput sequenced. The authors performed quality control and co-assembly of reads prior to binning. They then reconstructed MAGs and focused subsequent analyses on high-quality, non-redundant MAGs meeting thresholds of >50% completeness and <5% contamination. Metatranscriptomic reads (after rRNA depletion) were mapped back to a comprehensive gene catalog to quantify gene expression levels. This approach is robust and ensures that even genes from organisms not fully captured in MAG bins are counted in expression analyses. Overall, the laboratory and computational procedures for obtaining and processing the omics data are complete and state-of-the-art, consistent with previously published deep-sea metagenomic studies. The data quality appears sufficient to support the conclusions. Yet, the authors retained genomes with >50% completeness (and <5% contamination). By conventional standards >50% would be considered medium-quality draft genomes rather than high-quality. Many of the MAGs used for functional analysis could be missing nearly half their genes. This raises a caution:

when they report, for example, that “71 MAGs were incapable of generating required electron acceptors (lacking nitrite or sulfate-producing pathways)”, some MAGs might appear to lack a pathway simply because that part of the genome wasn’t recovered. If a MAG is 60% complete, the absence of a nitrification gene could be due to incomplete assembly rather than true absence in the living organism.

It would strengthen confidence if the authors indicate how many of those 71 MAGs were near-complete vs. just above 50%, or if they performed any cross-check (for example, searching unbinned contigs for those functions). Additionally, co-assembling all samples could, in theory, create chimeric bins if closely related strains differ between sites. The manuscript doesn’t detail the binning method or any manual curation steps. This is a standard practice but requires in depth explanation.

Response: We thank the reviewer for this thoughtful and important comment. We agree that genome incompleteness can potentially lead to underestimation of certain metabolic pathways, and that this limitation needs to be carefully considered when interpreting genome-resolved functional analyses.

In the revised manuscript, we have substantially improved our binning strategy to enhance MAG quality and to reduce the risk of chimeric bins. Specifically, assemblies were performed separately for each site, and all quality-controlled reads from samples within the same site were mapped

back to the assembled contigs to generate a contig coverage matrix. Based on recent benchmarking studies, we then applied multiple binning approaches (MetaBinner, SemiBin, and COMEBin) to generate primary MAG sets[1]. The use of site-specific assemblies combined with multiple complementary binning tools substantially improves MAG completeness and reduces binning artifacts associated with strain heterogeneity.

We acknowledge that incomplete MAGs may cause some functional genes to be missed, particularly for pathways inferred from gene absence. However, for large-scale genome-resolved analyses, a completeness threshold of ≥50% and contamination ≤5% is widely adopted in the field and represents a balance between genome recovery and dataset coverage[2,3]. We have therefore retained this criterion to ensure sufficient representation of community diversity. Regarding the suggestion to search unbinned contigs for missing functions, we chose not to assign unbinned contigs to existing MAGs. Such assignments require rigorous validation to ensure correct genome affiliation and to avoid introducing chimeric sequences. For a dataset of this scale, this approach is not readily tractable and could introduce additional uncertainty. We now explicitly clarify these limitations and methodological choices in the revised manuscript to ensure transparent interpretation of the results.

The revised methods were described in the supplementary Note 4 (file “2 supporting information” Line 132-140)

The downstream bioinformatic analyses in the manuscript are extensive and generally appropriate. The authors leveraged their annotated MAGs and gene expression data to infer metabolic capacities and active pathways in the community. However, a challenge is the limited sample size (n=9 sediment samples). The authors set a relatively high Pearson r threshold (≥0.6) for edges in the network, which is a reasonable filter, but significance testing with n=9 has low power. There is a risk of both false positives and false negatives in the network: some observed correlations might be spurious (driven by unmeasured factors or the compositional nature of relative abundance data), while some real interactions might not register due to the small sample size. The authors did mitigate this by requiring that MAGs appear in ≥90% of samples to be included. Yet, the interpretation should be softened and presented as hypothesis-generating rather than as a proof. Correlation does not equal causation. A brief note on how they normalized abundance data would be also useful.

Response: Thanks for your valuable comments. We fully acknowledge the limitation of the small sample size used in the previous version of our manuscript. (1) To address this concern, we have now substantially expanded our dataset. Specifically, we incorporated publicly available metagenomic data from hadal sediment samples in the Kermadec Trench, and we obtained newly sequenced data from additional samples provided

by our new collaborator, Dr. Huahua Jian. As a result, our study now includes 42 metagenomic datasets. This number of metagenomic datasets is sufficient for constructing a robust microbial co-occurrence network[1,2]. (2) We now use Spearman correlation to construct co-occurrence networks. And use Random Matrix Theory approach to determine the proper threshold. (3) For MAG selection, our criteria require that a MAG appears in at least 20% of all samples and reaches a relative abundance of at least 0.5% in at least one sample. By default, the input data are RPKM values of MAGs derived from each metagenomic dataset.

Details of the data description can be found in Lines 390–394, and the co-occurrence network construction methods are described in Lines 408–421.

There are few weaknesses also on electron microscope analyses. One minor concern is the abundance and significance of MTB: magnetotactic bacteria constitute a relatively small fraction of the community. The TEM images come from a concentrated search or enrichment. It's not stated how many cells or fields of view were examined to find the magnetosome-containing bacteria. If only a handful of cells were observed, one could question how representative or influential they are in situ.

Response: Thanks for your questions. Considering these limitations and related weaknesses noted by other reviewers, this revision has removed all description and discussion about MTB.

The impact of artifacts for this specific studied could be limited, but an explanation on how they handled or at least acknowledge this issue would be appropriate in a revision. The authors infer cross-feeding of electron acceptors based on absence/presence of genes, but they have not directly carried out measurements. The manuscript would benefit from explicit acknowledgment of this and perhaps suggestions for future experimental validation.

Response: Thanks for your comments. The constraints of our approach and their implications for data interpretation are now explicitly outlined in Lines 379–382 of the revised manuscript.

A central question motivating this work is why microbial biomass and activity in the trench are higher than expected for such an extreme, oligotrophic environment. The authors propose a compelling mechanistic interpretation: extreme pressure itself drives microbes to adopt versatile redox strategies that sustain high metabolic activity despite low oxygen. The mechanistic story is largely consistent with the data. The authors' multi-omic evidence clearly demonstrates diverse metabolic capabilities in the community: genes for aerobic respiration and anaerobic respiration were found actively expressed. The authors rightly highlight that MTB have been overlooked in deep-sea sediments and raise the possibility that

MTB are integral to element cycling in trenches. The main caveat is that much of this remains inference without direct experimentation. For example, the manuscript assumes that because transcripts were captured in situ, the high metabolic gene expression is truly happening at depth. However, could any of the "high activity" signals still be influenced by the sample handling? If RNAlater did not instantly fixed? The authors did not detect or mention any obvious artifacts.

Response: Thanks for your question. We have strengthened the methodological justification for our *in situ* preservation strategy by citing recent evidence demonstrating that in situ fixation can effectively minimize transcriptomic distortions caused by depressurization during sample recovery[1]. Specifically, He et al. compared multiple deep-sea sampling/preservation approaches and showed that in situ fixation better preserves community composition and transcriptional profiles relative to non–in situ approaches, highlighting the importance of immediate preservation for capturing in situ gene expression more accurately. Accordingly, we have revised the Methods (Lines 385-390) to clarify our fixation procedure and have emphasized this point in the Results (Lines 105–107).

1. He, Y., Zhang, H., Baltar, F. & Wang, Y. Transcriptional Difference of Deep-Sea Microorganisms under Different Sampling Methods. Environ. Sci. Technol. 59, 11653–11665 (2025).

Another uncertainty is how universal or large the MTB effect is. With MTB-related reads being a few percent of the community, can we be sure their vertical shuttling substantially fuels the rest of the microbes? The co-occurrence links imply many degraders could benefit from MTB, but it's not direct proof.

Response: Thanks for your questions. Considering these limitations and related weaknesses noted by other reviewers, this revision has removed all description and discussion about MTB.

The authors' interpretations would also be strengthened by acknowledging alternative explanations for high activity: for instance, simply the higher input of organic matter (as noted) and possibly physiological adaptations like piezolytes or specialized enzymes could contribute to resilience under pressure. The focus on redox coupling is interesting, but a broader context of hadal adaptation (e.g. pressure-resistant enzyme structures, membrane adaptations, etc.) is beyond this study's scope and is rightly left to citations.

Response: Thanks for your suggestions. Thanks for your suggestions. We have now cited relevant previous studies in the Discussion (Lines 266–271) to expand our description of microbial piezotolerance/pressure-adaptation traits. We also explicitly note in Lines 369–371 that the focus

of this study is the oligotrophic-adaptation strategies beyond pressure adaptation.

Some key conclusions (e.g., metabolic cooperation between organisms, the extent of MTB's role) are based on correlation and association rather than direct measurement. This is a natural limitation of environmental 'omics studies, but it means causality is inferred, not proven. A reader should keep in mind that alternative interpretations of the correlations are possible (though the authors' interpretations are well-reasoned).

Response: Thanks for your suggestions. The constraints of our approach and their implications for data interpretation are now explicitly outlined in Lines 379–382 of the revised manuscript.

Using MAGs with as low as 50% completeness could omit genes, so any conclusions that certain pathways are absent should be cautious. There is uncertainty in declaring what a microbe "cannot do" when its genome is partial, better to stick on what they can do.

Response: Thanks for your suggestions. we now focus our functional interpretation on positively supported evidence, specifically the genes that are present and actively transcribed in each MAG. Accordingly, we updated the relevant text and analyses to emphasize transcriptionally active metabolic genes as the basis for inferring MAG functions (e.g., Fig.

3A and Supplementary Table 4). This revision ensures that our conclusions highlight what these organisms are demonstrably capable of under the sampled conditions, rather than inferring the absence of functions from partial genome recovery.

Small errors can be easily addressed (such as referring to “Mariana Trench” instead of Kermadec in the Methods).

Response: We have corrected in Line 385. Thanks!

In conclusion, this manuscript presents sound, well-supported findings and employs a robust, cutting-edge methodological framework suitable for publication after appropriate revision. The core conclusions, that hadal trench microbes use versatile redox-coupling mechanisms (including possible magnetotactic shuttling) to maintain high metabolic activity under extreme pressure, are both novel and important for the field of deep-sea microbial ecology.

Reviewers' comments:

Reviewer #1 (Remarks to the Author):

The authors have done a good job addressing my comments overall.

However, there is still insufficient discussion of the limitations of their study. They mention in their response that "We now explicitly outline the limitations of genome completeness, sample size, and the inference-based nature of our metabolic conclusions (Lines 379-382)." I do not see that actually added into their manuscript, and lines 379-382 do not match that claim. This needs to be added clearly and extensively. A single sentence would be insufficient: a proper discussion of the limitations is needed.

Responses:

We thank the reviewer for this important comment. We agree that the limitations of the study were not sufficiently discussed in the previous version, and that our earlier response overstated the extent to which these limitations had been addressed in the manuscript. To resolve this issue, we have now added a dedicated paragraph in the Discussion section, immediately before the concluding paragraph, to more clearly discuss the major constraints of our study. Specifically, we now address the limitations associated with incomplete MAGs. We also clarify that, in the absence of paired geochemical measurements from the same sediment horizons, our results cannot directly quantify in situ redox conditions, electron acceptor availability, or biogeochemical fluxes. We believe this revision provides a more balanced interpretation of the data and more clearly defines the scope of our conclusions. This revision has been added to Lines 368-380.

Using publicly available data is valid, but the authors should also

consider to what degree technical differences between the samples from different sources could be driving the results. This should be explicitly explored when evaluating the key findings. E.g., is there a different proportion of samples from different studies in the slope vs. bottom samples? This would mean your signal was confounded with methodology if so, and should definitely be clearly shown one way or another.

Responses:

We thank the reviewer for raising this important point. We agree that potential technical differences among datasets from different sources should be explicitly evaluated.

1) In our dataset, the slope group included 22 samples, of which 15 were from public datasets, whereas the bottom group included 20 samples, of which 11 were from public datasets. Thus, public samples accounted for 68.2% of the slope samples and 55.0% of the bottom samples. The proportion of public versus newly generated samples did not differ significantly between the slope and bottom groups Fisher's exact test, $p = 0.53$.
2) To further evaluate whether sample source or associated technical differences could drive the observed community patterns, we performed an NMDS analysis based on sample source Supplementary Fig. 2b. The adonis test showed that differences among sample sources were not statistically significant $p = 0.06$. In contrast, the separation between slope and bottom samples was significant adonis, $p < 0.001$. These results suggest that the major community difference observed between slope and bottom samples was unlikely to be primarily driven by sample source or methodological differences.

We have now added this analysis and clarification to the revised manuscript (Lines 124-129, Supplementary Fig. 2b).

Also note the typo "quatatum" on line 382.

Responses:

We corrected the typo “quatatum” on line 398.

Reviewer #2 (Remarks to the Author):

I thank the authors for addressing my main comment regarding the magnetotactic bacteria and for restructuring the Results section accordingly. I have no further comments on this manuscript within my area of expertise.

Responses:

We sincerely thank the reviewer for their positive evaluation and constructive comments, which have helped improve the manuscript.

Reviewer #4 (Remarks to the Author):

I was asked to participate in this review process as a mediator, following unavailability of reviewer 3 for a review of the revised manuscript. As such, I have only considered whether the authors' responses clearly address the reviewer's comments.

Overall I would say this is the case, with the authors taking pains to increase the statistical power of their analysis, clarifying methods and removing a debated section. A few minor problems remain in my opinion:

- L379-382: The authors claim that this sentence "explicitly outline the limitations of genome completeness, sample size, and the inference-based nature of our metabolic conclusions" and highlights "the constraints of their approach and their implications for data interpretation", which does not appear to be the case. The sentence also seems to not make sense. I would suggest rephrasing and properly discussing the limitations of omics approaches without accompanying geochemistry data instead of simply suggesting avenues for further studies.

Responses:

We thank the reviewer for pointing out the lack of clarity in the previous sentence and for emphasizing the need to properly discuss the limitations of omics-based interpretations without accompanying geochemical data. We agree with this concern. In the revised manuscript, we have rephrased this section and expanded it into a dedicated limitation paragraph in the Discussion section. Rather than simply suggesting future directions, the revised text now explicitly explains how the absence of paired geochemical measurements limits our ability to directly assess porewater chemistry, redox gradients, electron acceptor availability, and

process rates. We also clarify that gene content and transcript abundance provide evidence of metabolic potential and transcriptional activity, but cannot by themselves establish direct links to in situ redox conditions or quantitative biogeochemical fluxes. This revision has been added to Lines 368-380.

- L385-390: In the response to the reviewer, the authors state that RNAlater was injected into the sediment cores before sealing them in situ. On the other hand the manuscript states “Surface sediments (0 ~ 10 cmbsf) were collected and immersed in RNAlater in situ” which is contradictory. Please fix this discrepancy and precisely report how the samples were collected, the amount of RNAlater that was injected or used for immersion, etc…

Responses:

We agree that the previous wording was ambiguous. We have revised the description to clarify the actual *in situ* fixation procedure. Specifically, surface sediments were first collected using a reusable insertion sampling tube and transferred into the upper chamber of a deep-sea sediment RNA fixation sampler. The lower chamber was preloaded with 360 ml RNAlater and separated from the upper chamber by a sealing membrane. After the sampler lid was closed, the manipulator actuated a pressure rod to release the RNAlater into the upper chamber, where it mixed with the sediment and fixed microbial RNA *in situ*. This revision has been added to Lines 403-409.

- L393: The correct citation for 51 (metagenomes produced from expedition TAN1711 to the Kermadec trench) should be Trouche et al., 2023 (https://doi.org/10.1038/s43705-023-00341-6). Although the current citation (Schauberger et al., 2024) analyses data

deposited in the same ENA project, it is Trouche et al. that describes the metagenomes and metadata used here.

Responses:

We thank the reviewer for pointing this out. We have corrected Reference 51 and now cite Trouche et al. (2023) as the appropriate source for the metagenomes. This revision has been added to Line 416.

Despite these minor remaining points, the authors have produced a rigorous and interesting study that will be a valuable contribution to our understanding of microbial life in hadal sediments.
